# NSUN2 Promotes Cancer Immune Evasion via Its Moonlighting Function Acting on Metabolic Reprogramming

**DOI:** 10.34133/cancomm.0042

**Published:** 2026-08-19

**Authors:** Baoxiang Chen, Yanrong Deng, Xiang Zhai, Peiyun Wang, Kunming Lei, Siyuan Yin, Quanjiao Chen, Qun Qian, Jinfang Zhang, Xianghai Ren, Jianhong Zhao, Congqing Jiang

**Affiliations:** ^1^ Hubei Key Laboratory of Intestinal and Colorectal Diseases, Zhongnan Hospital of Wuhan University, Wuhan, Hubei, P. R. China.; ^2^ Clinical Center of Intestinal and Colorectal Diseases of Hubei Province, Zhongnan Hospital of Wuhan University, Wuhan, Hubei, P. R. China.; ^3^ Department of Colorectal and Anal Surgery, Zhongnan Hospital of Wuhan University, Wuhan, Hubei, P. R. China.; ^4^ Low Rectal Cancer Diagnosis and Treatment Center, Zhongnan Hospital of Wuhan University, Wuhan, Hubei, P. R. China.; ^5^ Rosalind and Morris Goodman Cancer Institute, McGill University, Montreal, QC, Canada.; ^6^ Department of Colorectal and Anal Surgery, Jingzhou Hospital Affiliated to Yangtze University, Jingzhou, Hubei, P. R. China.; ^7^ Key Laboratory of Special Pathogens and Biosafety, Chinese Academy of Sciences, Wuhan, Hubei, P. R. China.; ^8^ Frontier Science Center of Immunology and Metabolism, Medical Research Institute, Wuhan University, Wuhan, Hubei, P. R. China.; ^9^ State Key Laboratory of Metabolism and Regulation in Complex Organisms, College of Life Sciences, Taikang Center for Life and Medical Sciences, Wuhan University, Wuhan, Hubei, P. R. China.

## Abstract

**Background:** Although cancer immunotherapies have revolutionized cancer treatment, a substantial proportion of patients remain unresponsive. Elucidating the molecular mechanisms underlying tumor immune evasion and identifying key regulators are essential for improving immunotherapy efficacy. NOP2/Sun RNA methyltransferase 2 (NSUN2) exhibits widespread mutations across pan-cancer cohorts. This study aimed to delineate the noncanonical functions of NSUN2 in cancer immune modulation and explore its potential as a therapeutic target for cancer immunotherapy. **Methods:** Multiple cancer cells expressing catalytically inactive NSUN2 mutants were generated and subjected to in vitro functional assays and in vivo studies in immunocompetent mouse models to evaluate their effects on tumor growth and antitumor immunity. Integrative multi-omics analyses, including transcriptomics, metabolomics, and mass spectrometry, were performed to elucidate the molecular mechanisms underlying NSUN2-mediated immune evasion. A proteolysis-targeting chimera (PROTAC) system was developed to achieve targeted degradation of NSUN2, and the clinical relevance of NSUN2 expression in predicting immunotherapy responses was assessed using institutional and public datasets. **Results:** The enzymatically inactive NSUN2 mutant had minimal effects on tumor cell proliferation in vitro but markedly promoted tumor immune evasion in vivo. Multi-omics analyses revealed that NSUN2 induced metabolic reprogramming and elevated succinate levels, which suppressed cyclic GMP-AMP synthase (cGAS)–stimulator of interferon genes (STING) signaling in tumor-associated macrophages (TAMs), thereby remodeling the tumor immune microenvironment and promoting M2-like TAM infiltration. Mechanistically, NSUN2 interacted with GATA-binding protein 3 (GATA3) through its methyltransferase domain, relieving GATA3-mediated transcriptional repression of succinate-CoA ligase GDP/ADP-forming subunit α and β genes (SUCLG1 and SUCLG2), leading to succinate accumulation. A newly developed NSUN2-targeting PROTAC demonstrated therapeutic efficacy and safety in combination with cancer immunotherapy. Clinically, low NSUN2 expression was associated with improved immunotherapy responses and survival. **Conclusions:** Taken together, these findings revealed a noncanonical role of NSUN2 in reshaping the tumor immunosuppressive microenvironment, positioning NSUN2 as a pivotal repressor of cancer immunity and a promising immunotherapeutic target.

## Background

Immunotherapy is among the most groundbreaking developments that have witnessed remarkable success in the treatment of various cancers; however, only approximately 10% to 30% of patients respond to it [1]. Immune checkpoint blockade (ICB) therapies targeting programmed cell death 1 receptor (PD-1) or its ligand programmed cell death ligand 1 (PD-L1) have demonstrated remarkable clinical benefits in multiple cancer types; yet, durable responses have been observed in only a limited subset of patients, and the mechanisms underlying these low response rates remain unclear [2]. Thus, elucidating the specific mechanisms of cancer immune evasion is essential, and designing combination immunotherapies is a promising strategy to overcome resistance and enhance the efficacy of current ICB treatments. In previous studies, we found that tripartite motif containing 21 (TRIM21) [3]; ubiquitin specific peptidase 5 (USP5) [4]; USP8 [5]; UFM1 specific ligase 1 (UFL1) [6]; OTU deubiquitinase, ubiquitin aldehyde binding 1 (OTUB1) [7]; and USP2 mediate posttranslational modifications of key immune checkpoints [8], including PD-1, PD-L1, and CD47, whereas methyltransferase 3 (METTL3) and phosphorylated CTD interacting factor 1 (PCIF1) modulate RNA modifications of *PD-L1* and ferroptosis-related suppressor genes [9,10], building on our long-standing research focus and expertise, highlighting their collective role in regulating cancer immunity.

Epigenetic mechanisms are crucial not only for the initiation and progression of cancer but also for regulating the activation, differentiation, and effector functions of immune cells [11]. Several key immune-related molecules, such as granzyme B, interferon (IFN)-γ, interleukin (IL)-2, IL-12, forkhead box P3 (FoxP3), stimulator of interferon genes (STING), and immune checkpoint proteins, including PD-1, cytotoxic T-lymphocyte-associated protein 4 (CTLA-4), T-cell immunoglobulin and mucin domain-containing protein 3 (TIM-3), lymphocyte activation gene 3 (LAG-3), and T-cell immunoreceptor with Ig and ITIM domains (TIGIT), expressed in immune and tumor-associated stromal cells, are epigenetically regulated, making epigenetic-modulating therapies a promising strategy for remodeling the tumor microenvironment (TME) [11]. Among the epigenetic transcription-associated NOP2/Sun RNA methyltransferases (NSUNs), the methyltransferase NSUN2 remains enigmatic despite being identified more than 30 years ago [12–14]. A pivotal study revealed that glucose, as a cofactor, binds to NSUN2 at amino acids 1 to 28 to promote NSUN2 oligomerization and activation in cancer cells, thereby promoting tumorigenesis [12]. Beyond its role in cancer, NSUN2 has been implicated in a range of nonmalignant pathological conditions, such as colitis [13] and diabetic retinopathy [14]. In a previous study, we identified the classical role of NSUN2-mediated 5-methylcytosine (m^5^C) modification in driving the progression and metastasis of colorectal cancer (CRC) via the NSUN2/enolase 1 (ENO1) positive feedback loop [15]. However, this study focusing on the classical role of NSUN2 in tumorigenesis primarily used in vitro systems or immunodeficient models [15], leaving its nonclassical functions in TME remodeling largely unexamined. Inflammatory cells are essential components of the TME ecological niche, with macrophages constituting a predominant component of the leukocyte infiltrate, present at varying abundances across nearly all tumor types [16]. Tumor-associated macrophages (TAMs) display high phenotypic plasticity in the TME [16], and they are categorized into proinflammatory TAM1 and anti-inflammatory TAM2 subtypes, with distinct markers, metabolic features, and gene expression profiles [17]. Although TAMs predominantly adopt a TAM2-like protumorigenic phenotype in the TME [18], their intrinsic plasticity enables functional repolarization toward a tumor-suppressive TAM1 state, and targeting this phenotypic conversion is critical for boosting antitumor immunotherapeutic efficacy.

Distinct metabolic rewiring, including alterations in glycolysis, the tricarboxylic acid (TCA) cycle activity, and fatty acid or glutamine metabolism, reshapes macrophage polarization and underpins their functional specialization within the TME [19]. Elucidating the metabolic mechanisms that regulate TAMs and targeting this pathway may help overcome immunosuppression and enhance antitumor immunity. Nevertheless, whether epigenetic reprogramming governs TAM metabolic rewiring and subsequent phenotypic polarization remains poorly defined. To date, the immunomodulatory roles of NSUN2 in the TME remain are largely uncharacterized. Of particular note, the current literature lacks evidence on whether NSUN2 facilitates tumor immune evasion through noncatalytic pathways independent of its canonical RNA methyltransferase activity. Addressing these unresolved questions is critical to fully elucidating the epigenetic and metabolic regulatory mechanisms controlling TAM-mediated immune suppression and improving the understanding of cancer immune regulation. Herein, we propose an innovative multidimensional research strategy by integrating immunocompetent and immunodeficient mouse models, multi-omics profiling, and biochemical assays including co-immunoprecipitation (co-IP) and mass spectrometry (MS). Combining rigorous in vitro and in vivo functional experiments, we specifically explored the unprecedented noncatalytic role of NSUN2 in governing TCA cycle reprogramming, macrophage polarization, and TME remodeling. Importantly, this study further investigated the translational potential of NSUN2 targeting to sensitize ICB therapy, offering novel mechanistic insights and promising therapeutic targets for clinical tumor immunotherapy.

## Materials and Methods

### Human samples

Thirty human CRC and 30 breast cancer (BC) tissues, along with matched adjacent normal tissues, were obtained from histologically confirmed patients at the Zhongnan Hospital of Wuhan University with informed consent between September 2021 and June 2025, under a protocol approved by the hospital’s Ethics Committee (approval number: 2025094K). Patients with CRC or BC who received immune checkpoint inhibitor therapy (sintilimab, tislelizumab, or camrelizumab) were classified as responders if they achieved a complete response (CR) or partial response (PR), and as nonresponders if they exhibited stable disease (SD) or progressive disease (PD). CR was defined as the disappearance of all target lesions; PR as at least a 30% decrease in the sum of diameters of target lesions, taking as reference the baseline sum diameters; PD as at least a 20% increase in the sum of diameters of target lesions, with an absolute increase of at least 5 mm, or the appearance of new lesions; and SD as neither sufficient shrinkage to qualify for PR nor sufficient increase to qualify for PD.

### Cell lines and cell culture

Human embryonic kidney (HEK293T) and mouse colorectal carcinoma (MC38) were obtained from the Jinfang Zhang Laboratory (Wuhan University), whereas human colorectal carcinoma (SW480), mouse breast carcinoma (4T1), human monocytic (THP-1), and mouse macrophage (RAW264.7) cell lines were purchased from the American Type Culture Collection. Cell line identity was verified via short tandem repeat profiling, and mycoplasma contamination was excluded using the MycoAlert Mycoplasma Detection Kit (Lonza). Cells were cultured in Dulbecco’s modified Eagle medium (DMEM; Gibco) or Roswell Park Memorial Institute 1640 medium (RPMI 1640; Gibco) supplemented with 10% heat-inactivated fetal bovine serum (FBS; Gibco) and 1% penicillin–streptomycin (Beyotime) under standard conditions (37 °C, 5% CO_2_). For pharmacological inhibition experiments, cells were incubated with 5 μmol/l MY-1B (NSUN2-targeting m^5^C methyltransferase inhibitor; HY-158301, MedChemExpress) or 50 μmol/l pyrrothiogatain (GATA-binding protein 3 [GATA3] inhibitor; HY-138113, MedChemExpress] for 48 h.

### Plasmids, transfection, and virus infection

The human expression constructs pLV3-CMV-3Flag-*NSUN2*, succinate-CoA ligase GDP/ADP-forming subunit α and β genes (*SUCLG1* and *SUCLG2*), along with the packaging plasmids psPAX2 and pMD2.G, were purchased from MiaoLing Biology, whereas the human constructs pLV3-CMV-HA-*GATA3*, pLV3-CMV-3Flag-*NSUN2*^MUT^, and pLV3-CMV-*NSUN2*-sgRNA-Cas9, together with the mouse constructs pLV3-CMV-*Nsun2*, *Gata3*-sgRNA-Cas9, pLV3-CMV-HA-*Gata3*, and pLV3-CMV-3Flag-*Nsun2*^MUT^, were synthesized by Sangon Biotech. In both human and mouse *NSUN2* mutants, cytosine (C) at positions 271 and 321 was mutated to adenine (A), resulting in the loss of the m^5^C methylation function. HEK293T cells that reached 60% to 80% confluence were transfected with the indicated plasmids using Lipo8000 (C0533, Beyotime) in optimized minimal essential medium (Opti-MEM, 31985-070, Gibco). The cells were harvested 36 to 48 h after transfection for downstream assays, including western blotting (WB), IP, and reverse transcription quantitative polymerase chain reaction (RT-qPCR). To generate stable cell lines via lentiviral packaging, HEK293T cells were transfected in 6-cm dishes with 2 μg of the control or target gene plasmid, 2 μg of psPAX2, and 1 μg of pMD2.G. After 36 to 48 h, the virus-containing supernatant was collected, filtered through a 0.45-μm syringe filter, and used to infect target cells. Target cells at 30% to 40% confluence were then infected with viral supernatants supplemented with 8 μg/ml polybrene (107689, Sigma-Aldrich). Following infection, the cells were treated with puromycin for 36 to 48 h. The guide RNA sequences used in this study are presented in Table S1.

### Animal models

All animal procedures were approved by the Animal Care Committee of Wuhan Institute of Virology, Chinese Academy of Sciences (CAS) (approval number: WIVA04202301). In this study, we utilized 6-week-old female C57BL/6 (GemPharmatech), BALB/c mice (GemPharmatech), NOD-*Prkdc*^scid^-*Il2rg*^null^ (NSG, GemPharmatech), and NOD.Cg-*Prkdc^scid^Il2rg^tm1Sug^*/ShiJic mice (NOG, Beijing Vital River Laboratory Animal Technology). The succinate receptor 1 ‌(*Sucnr1*) knockout (*Sucnr1*^−/−^) mice were obtained from Cyagen Biosciences and generated using the CRISPR–Cas9 method. To assess the role of the immune system in *Nsun2*^MUT^-mediated tumor regulation, 2 × 10^5^ empty vector (EV)- or *Nsun2*^MUT^-expressing MC38 or 4T1 cells were subcutaneously injected into immunodeficient NSG and immunocompetent C57BL/6 or BALB/c mice. For xenograft experiments, EV or *NSUN2*^MUT^ SW480 cells (1 × 10^6^ cells in 100 μl of phosphate-buffered saline [PBS]) were subcutaneously injected into NOG mice. After tumor cell inoculation, the mice were randomly assigned to 2 treatment groups: PBS or human peripheral blood mononuclear cells (PBMCs; 1 × 10^7^ cells).

The azoxymethane (AOM)/dextran sodium sulfate (DSS)-induced CRC model was established in 8-week-old C57BL/6 mice. The mice received a single intraperitoneal injection of AOM (10 mg/kg; A5486, Sigma-Aldrich), followed 1 week later by 2.5% DSS (160110, MP Biomedicals) in drinking water for 7 d, followed by regular water for 14 d. This cycle was repeated twice, and the mice were euthanized by cervical dislocation on day 85 for tumor assessment. For experiments with the adenovirus system, mice were treated as described above and subsequently administered adenovirus-*Nsun2*^MUT^ or adenovirus-EV (5 × 10^8^ pfu per mouse) via tail vein injection every 2 weeks for 12 weeks.

For toxicity assessment, male and female nontumor-bearing C57BL/6 mice were treated with NSUN2 PROTAC-1 (NP-1) at 2 mg/kg for 2 doses over the first 10 d, followed by 5 mg/kg for 2 additional doses during the subsequent 10 d, for a total of 4 administrations. For the NP-1 and anti-PD-L1 antibody treatment experiments, mice received intraperitoneal injections of NP-1 (2 mg/kg) once every 5 d on days 0, 5, 10, and 15 and/or 200 μg of anti-PD-L1 antibody (BE0101, Bioxcell) once every 4 d on days 7, 11, 15, and 19, administered either alone or in combination (4 doses per agent). Mice were euthanized by cervical dislocation on day 28 for tumor assessment. All animals were housed in a specific-pathogen-free facility at the Wuhan Institute of Virology, CAS, under controlled temperature and 50% to 60% relative humidity.

### Cell proliferation assay

Cell proliferation was determined using Cell Counting Kit-8 (CCK-8, C0037, Beyotime), according to the manufacturer’s protocol. Briefly, cancer cells (1 × 10^3^ per well) were seeded into 96-well plates and incubated overnight. At 24, 48, and 72 h, 10 μl of CCK-8 solution was added to each well, followed by a 1-h incubation at 37 °C. Absorbance was measured at 450 nm using a microplate reader (Bio-Rad Laboratories, Hercules).

### Colony formation assay

Cells were seeded into 6-well plates at a density of 1,000 cells per well and cultured for 8 to 12 d until visible colonies were formed. The colonies were washed with PBS, fixed with paraformaldehyde for 30 min, and stained with 0.1% crystal violet for 15 min. After washing and air-drying, the colonies were counted and photographed by a high-resolution digital camera.

### IP, subcellular fractionation, and WB analysis

IP and WB analyses were conducted as previously described [20]. Cells were lysed in radioimmunoprecipitation assay (RIPA) buffer supplemented with phenylmethylsulfonyl fluoride (PMSF; P7626, Sigma-Aldrich) and phosphatase inhibitors (P0044, Sigma-Aldrich). For IP, cell lysates were incubated overnight at 4 °C with anti-Flag (HY-K0207, MedChemExpress) or anti-HA (HY-K0201, MedChemExpress) magnetic beads, followed by 3 washes with tris-buffered saline with Tween-20 (TBST) buffer and resolution via sodium dodecyl sulfate–polyacrylamide gel electrophoresis (SDS-PAGE). For IB analysis, cells subjected to different treatments were lysed in cell lysis buffer containing PMSF and phosphatase inhibitors. The antibodies used for IP and IB are listed in Table S2.

### RNA sequencing

MC38 tumors were established in 6-week-old C57BL/6 mice by subcutaneous injection of 2 × 10^5^ EV or *Nsun2*^MUT^ MC38 cells. Twenty-four days after inoculation, tumors were excised, minced, and enzymatically digested with collagenase IV (1 mg/ml) and DNase I (100 U/ml) at 37 °C for 30 min to generate single-cell suspensions. Cells were filtered through a 70-μm strainer. After washing, cells were stained with anti-mouse CD45 antibody and a viability dye for 30 min at 4 °C. Live single cells were gated, and mCherry^+^CD45^−^ cells were sorted by flow cytometry and collected for RNA sequencing (RNA-seq) analysis. Total RNA for RNA-seq was extracted from either MC38 tumor cells or RAW264.7 cells treated with or without 100 μmol/l succinate (Sigma-Aldrich) for 48 h, using TRIzol reagent (15596018, Invitrogen) according to the manufacturer’s instructions. The enriched messenger RNA (mRNA) was fragmented using a fragmentation buffer and reverse-transcribed into complementary DNA (cDNA) using the NEBNext Ultra RNA Library Prep Kit for Illumina (E7760L, New England Biolabs). Purified double-stranded cDNA fragments were end-repaired, A-tailed, and ligated to Illumina sequencing adapters. The ligation products were purified using AMPure XP beads (A63881, Beckman Coulter), and the resulting cDNA library was sequenced on an Illumina NovaSeq 6000 platform (Gene Denovo Biotechnology Co., Ltd.) according to the manufacturer’s protocol.

For RNA-seq data analysis, raw FASTQ reads were first processed using fastp (v1.0.1, https://github.com/OpenGene/fastp) to remove low-quality sequences and yield clean reads. The resulting clean reads were aligned to the *Mus musculus* mm10 reference genome using HISAT2 (v2.2.1, https://daehwankimlab.github.io/hisat2/download), followed by gene-level read quantification using featureCounts (v2.0.3, http://subread.sourceforge.net/featureCounts.html) and subsequent differential expression analysis. Differential expression analysis was conducted using the DESeq2 package (v1.42.0) in R (v4.3.2, R Foundation for Statistical Computing). Genes with a *P* <0.05 and a fold change >2 or <0.5 were defined as differentially expressed genes (DEGs). Based on the hypergeometric distribution, Gene Ontology and Kyoto Encyclopedia of Genes and Genomes (KEGG) pathway enrichment analyses of DEGs were performed using the clusterProfiler package (v4.10.0) in R to identify significantly enriched terms. Gene set enrichment analysis (GSEA) was carried out using the clusterProfiler package with a predefined gene set, and genes were ranked according to the magnitude and direction of differential expression between the 2 sample groups.

### TCA cycle metabolite analysis

Targeted metabolomics of TCA cycle intermediates was performed on an ultra-performance liquid chromatography coupled to triple quadrupole mass spectrometry platform (ACQUITY UPLC-Xevo TQ-S, Waters) by Metabo-Profile Biotechnology. A total of 2 × 10^6^ mCherry^+^CD45^−^ cells were sorted by flow cytometry, thawed on ice, and extracted with 120 μl of ice-cold methanol (MeOH) containing internal standards. After ultrasonic disruption (JY92-IIN), homogenates were centrifuged at 18,000 ×*g* for 20 min at 4 °C. Then, 30 μl of the supernatant was derivatized with 20 μl of the metabolite derivatization reagent (3-nitrobenzhydrazide hydrochloride, ZTO-N0232, Tokyo Chemical Industry) at 30 °C for 60 min with shaking (1,450 rpm), followed by the addition of 330 μl of 50% MeOH and centrifugation at 4,000 ×*g* for 30 min at 4 °C. Chromatographic separation was achieved on a BEH C18 column (100 mm × 2.1 mm, 1.7 μm) at 40 °C using a 0.1% formic acid gradient (water/MeOH) at 0.3 ml/min. Data were acquired in electrospray ionization ± modes, processed with MassLynx v4.1 (Waters Corporation) for quantification against standard curves, and statistically analyzed with the iMAP software (v1.0, Metabo-Profile). Quality controls (QCs) included pooled QC samples inserted every 10 injections and reagent blanks to ensure reproducibility and minimize matrix effects.

### IP coupled with MS (IP–MS) for identifying NSUN2-interacting protein

HEK293T cells transfected with either Flag-*NSUN2*^MUT^ or an EV were treated with 10 μmol/l MG132 (S1748, Beyotime) for 12 h prior to harvest and then lysed in extraction buffer for co-IP (50 mmol/l tris, pH 7.5, 0.5% NP-40, and 120 mmol/l NaCl) supplemented with protease and phosphatase inhibitors. Cell lysates were incubated with anti-Flag agarose beads overnight at 4 °C on a rotating platform. After incubation, the beads were washed 4 times with nonionic detergent, ethylenediaminetetraacetic acid (EDTA), tris, NaCl buffer (NETN) buffer to eliminate nonspecific binding. The immunoprecipitated complexes were then resuspended in 100 μl of 1× loading buffer and boiled at 95 °C for 10 min. Thereafter, the samples were resolved via SDS-PAGE, and the immunoprecipitated complexes were submitted to SpecAlly Life Technology Co., Ltd. for MS analysis. Protein abundance was quantified based on the detected peptide counts for each identified protein.

### RT-qPCR

Total RNA was extracted using the HiPure Total RNA Mini Kit (R4111, Magen), and reverse transcription was performed using the ABScript III RT Master Mix for qPCR with gDNA Remover (R323-01, Vazyme). The resulting cDNA was mixed with primers, probes, and ChamQ Universal SYBR qPCR Master Mix (Q711-02, Vazyme), and quantitative real-time PCR was conducted on a Bio-Rad CFX Connect Real-Time PCR Detection System, with glyceraldehyde-3-phosphate dehydrogenase (*GAPDH*) serving as an internal reference gene. The relative expression was determined using the comparative cycle threshold (2^−ΔΔCT^) method. The primer sequences are listed in Table S1.

### Chromatin IP assay

Chromatin IP (ChIP) was performed following the manufacturer’s protocol (P2078, Beyotime) with a GATA3 antibody (66400-1-Ig, Proteintech) or NSUN2 antibody (20854-1-AP, Proteintech) or an immunoglobulin G (IgG) control. DNA was purified using the Omega DNA Purification Kit (D2500, Omega Bio-tek) and analyzed via agarose gel electrophoresis and RT-qPCR. Fold enrichment was calculated relative to the input DNA using the primer sequences listed in Table S1.

### RNA immunoprecipitation and qPCR

Native RNA immunoprecipitation (RIP) was performed as described previously, with minor modifications [21,22]. For RIP–qPCR, 1.5 × 10^7^ cells were transfected with the Flag-tagged target protein expression plasmid, followed by cross-linking, cell harvesting, and lysis. Insoluble debris was removed via centrifugation at 1,500 ×*g* for 10 min at 4 °C, and the clarified lysate was divided for overnight pull-down with either anti-Flag or IgG-conjugated magnetic beads. The beads were then incubated with the lysates and washed with RIPA buffer containing DNase I, followed by Proteinase K digestion. RNA was purified using the MicroElute RNA Clean-Up Kit (R6247-01, Omega Bio-tek). Equal volumes of input and immunoprecipitated RNA were reversely transcribed into cDNA, and the enriched RNA was quantified using qPCR. The data were normalized to the input and expressed as percentages of the input.

### Luciferase reporter assay

The luciferase reporter construct (pGL3-*SUCLG1*/*SUCLG2*) was generated by cloning the upstream regions of these genes that are bound by GATA3 into the pGL3 basic vector. The final products were verified by enzymatic digestion and Sanger sequencing. HEK293T cells (2 × 10^5^) were seeded 24 h prior to transfection. These cells were cotransfected using Lipo8000 in triplicate, with a pCMX-β-galactosidase (β-gal) plasmid used as an internal control for checking the transfection efficiency, along with pGL3-*SUCLG1*/*SUCLG2* and in the presence or absence of the expression vectors, pCDH-*NSUN2*^WT^, pCDH-*NSUN2*^MUT^, pCDH-*NSUN5*, or pCDH-*NSUN6*. pCDH-GFP was added to ensure that the total quantity of plasmid DNA was equal. The cells were harvested and lysed using a lysis buffer at 48 h posttransfection. Luciferase activity was determined using the Promega Luciferase Assay System (PRE1500, Promega) and β-gal activity was detected using the Galacto-Star β-Galactosidase Reporter Gene Assay System for Mammalian Cells (T1014, Thermo Fisher Scientific) according to the manufacturer’s protocols. The luminescence of firefly luciferase from the pGL3 basic reporter was normalized to that of β-gal.

For the cyclic GMP-AMP synthase (cGAS) luciferase assay, 2 × 10^5^ HEK293T cells were transfected with 1 μg of the pGL3-cGAS promoter luciferase vector, negative control plasmid, and 100 ng of the pRL-TK-Renilla luciferase plasmid. The cells were then treated with dimethyl sulfoxide (DMSO) or succinate, and after 48 h, luciferase activity was measured using the Dual-Luciferase Reporter Assay System (TM040, Promega). Firefly luciferase activity was normalized to renilla luciferase activity and expressed as fold change relative to the control. Luciferase experiments were performed independently with 3 technical replicates.

### Immunofluorescence analysis and multiplex immunofluorescence analysis

Immunofluorescence (IF) was performed as previously described [8,15]. Briefly, treated cells grown on confocal dishes were fixed with 4% paraformaldehyde, permeabilized with 0.1% Triton X-100, and blocked with 5% bovine serum albumin. Cells were incubated with primary antibodies overnight at 4 °C, followed by fluorophore-conjugated secondary antibodies. Nuclei were counterstained with 4′,6-diamidino-2-phenylindole (DAPI), and images were acquired using a confocal microscope (Leica STELLARIS 8). For multiplex IF of paraffin-embedded tissues, sections were deparaffinized, subjected to antigen retrieval, and sequentially incubated with primary antibodies and horseradish peroxidase-conjugated secondary antibodies, followed by tyramide signal amplification according to the manufacturer’s instructions. After each staining cycle, antibody stripping was performed before the next round. Slides were counterstained with DAPI and imaged using the same confocal system. The antibodies used are listed in Table S2.

### Isolation and quantification of mitochondrial DNA

Mitochondrial DNA (mtDNA) was isolated as previously described [20] and purified using the DNA Purification Kit (D2500, Omega Bio-tek). RT-qPCR was performed on whole-cell lysates and cytosolic fractions using nuclear DNA primers (18S and Tert) and mtDNA primers (D-loop and ND1), with mtDNA levels normalized to nuclear DNA.

### Flow cytometry

MC38 tumor tissues were cut into approximately 3-mm fragments and enzymatically digested in DMEM containing 1 mg/ml collagenase IV (C8160, Solarbio) and 0.1 mg/ml DNase I (EN0523, Thermo Fisher Scientific) at 37 °C for 30 min. The resulting cell suspension was passed through a 70-μm cell strainer and washed twice with staining buffer (PBS supplemented with 2% FBS and 1 mmol/l EDTA). Cells were then resuspended in staining buffer, incubated with the indicated antibodies at appropriate dilutions for 1 h at 4 °C in the dark, and subsequently subjected to flow cytometric analysis. For intracellular staining, cells were fixed and permeabilized with the eBioscience Intracellular Fixation and Permeabilization Buffer (88-8824-00, Invitrogen) for 20 min at room temperature in the dark. For intracellular cytokine staining, 1 × 10^6^ cells were stimulated with phorbol 12-myristate 13-acetate (50 ng/ml), ionomycin (500 ng/ml), and brefeldin A (5 μg/ml) for 3 h at 37 °C prior to fixation and staining. Cells were then washed twice with staining buffer and resuspended in staining buffer followed by antibody staining. Finally, the abundances of CD8^+^ T cells (CD45^+^CD3^+^CD8^+^), TAM1-like TAMs (CD45^+^CD11b^+^F4/80^+^CD86^+^iNOS^+^), TAM2-like TAMs (CD45^+^CD11b^+^F4/80^+^ARG1^+^CD206^+^), INFγ^+^CD8^+^ T cells (CD8^+^INFγ^+^), and GzmB^+^CD8^+^ T cells (CD8^+^GzmB^+^) were determined using flow cytometry (BD LSRFortessa cell analyzer; RRID: SCR_018655) and analyzed using FlowJo v10 (RRID: SCR_008520).

### Hematoxylin and eosin staining and multiplex immunohistochemistry

The mouse heart, lung, kidney, spleen, and liver tissues were fixed in paraformaldehyde for at least 12 h at room temperature and embedded in paraffin. The paraffin-embedded tissues were sectioned at 5-μm thickness and stained with hematoxylin and eosin (H&E; C0105S, Beyotime). Multiplex immunohistochemistry (mIHC) staining was conducted at Pinuofei Biological, following a previously reported method [20]. Briefly, tumor tissues from tumor-bearing mice and human CRC, BC, and adjacent normal tissues were collected, fixed in 4% paraformaldehyde overnight, transferred to 70% ethyl alcohol (EtOH), and embedded in paraffin. Microscopic images of H&E- and mIHC-stained sections were acquired using Pannoramic Scan (3DHistech). The slides were analyzed using the ImageJ software, with 3 fields examined per independent tumor tissue from each mouse or patient. Patients were stratified into high- and low-target-protein-expression groups based on the median expression value calculated from all 30 patient samples.

The details of the antibodies used for mIHC are listed in Table S2.

### Isolation of bone-marrow-derived macrophages

Bone-marrow-derived macrophages (BMDMs) were isolated from C57BL/6 mice and prepared as described previously [20]; they were cultured in RPMI 1640 medium supplemented with 10% FBS, 1% penicillin–streptomycin, and 50 ng/ml mouse macrophage colony-stimulating factor (RP01216, ABclonal) to induce macrophage differentiation over 7 d. On day 7, BMDMs were refreshed with RPMI 1640 medium and stimulated with cytokines: 200 ng/ml IFN-γ (PRP1138, Abbkine) and 100 ng/ml lipopolysaccharide (L4516, Sigma-Aldrich) for TAM1-like activation or with 20 ng/ml IL-4 (RP01161, ABclonal) for TAM2-like activation. For co-culture experiments, transfected MC38 cells (1 × 10^5^ cells/well) were seeded into 0.4-μm upper chamber inserts 1 d before co-culture. The next day, the inserts were transferred to 6-well plates pre-seeded with BMDMs (1 × 10^6^ cells/well). After 48 h of co-culture, the cells and supernatants were collected for RT-qPCR, flow cytometry, WB, or enzyme-linked immunosorbent assay (ELISA). For ELISA, culture supernatants were collected, centrifuged at 12,000 ×*g* for 10 min at 4 °C to remove debris, and analyzed using commercially available ELISA kits (Jianglai Biological) according to the manufacturer’s instructions. Cytokine concentrations were determined based on standard curves generated using recombinant standards.

### Co-culture assays

CD8^+^ T cells were isolated from the spleen of wild-type (WT) C57BL/6 mice using the CD8 T Cell Isolation Kit (480035, BioLegend) after red blood cell lysis. Purified CD8^+^ T cells were incubated for 24 h with plate-bound anti-CD3 (2 μg/ml; 100340, BioLegend) and soluble anti-CD28 (2 μg/ml; 102116, BioLegend) antibodies in complete RPMI 1640 medium supplemented with IL-2 (100 U/ml; 212-12, PeproTech). The activated CD8^+^ T cells were then co-cultured at a 1:1 ratio with WT or *Sucnr1*^−/−^ C57BL/6-derived BMDMs that had been pretreated with tumor-conditioned medium (TCM) derived from different MC38 cells for 36 h, followed by IFN-γ^+^ and Gzmb^+^ staining to evaluate the T-cell activation status.

### Quantitation of succinate

MC38, 4T1, and SW480 cancer cells were seeded at a density of 3 × 10^6^ cells per 10-cm culture dish in 10 ml of culture medium, followed by different treatments or infections. The culture medium was then collected, and succinate levels were quantified using the Succinate (Succinic Acid) Colorimetric Assay Kit (K649-100, BioVision) according to the manufacturer’s protocol. Subcutaneous tumors were excised from C57BL/6 mice, and 100 mg of tumor tissue was weighed and transferred to a 2-ml microcentrifuge tube for homogenization on ice. The homogenate was centrifuged at 14,000 ×*g* for 10 min at 4 °C to collect the supernatant, which was then analyzed for succinate levels using the Succinate Colorimetric Assay Kit at 450-nm absorbance.

### Hematology analysis

C57BL/6 mice subjected to different treatments were anesthetized with isoflurane or ketamine/xylazine to minimize pain and stress in accordance with Institutional Animal Care and Use Committee guidelines. Blood was collected by gently inserting a sterile microhematocrit capillary tube or micropipette into the retro-orbital sinus. Samples were prepared in microcentrifuge tubes containing PBS and 10 mmol/l EDTA to prevent coagulation. Blood indices, including white blood cell, red blood cell, platelet, and lymphocyte counts, were analyzed using an automated hematology analyzer (Element HT5, Heska).

### Chemical synthesis of the PROTAC molecule NP-1

The proteolysis-targeting chimera (PROTAC) molecule NP-1 was chemically synthesized by MedChemExpress. To a suspension of *N*-Boc-4-hydroxyaniline (CAS 54840-15-2 1.32 g, 6.29 mmol, 1.5 equiv) and K_2_CO_3_ (1.74 g, 12.6 mmol, 3.0 equiv) in DMF (20 ml) was added bromo-polyethylene glycol 5 (PEG5)-*t*-butyl ester (CAS 1415800-38-2, 1.80 g, 4.20 mmol, 1.0 equiv). The mixture was stirred at 100 °C for 16 h. After completion (liquid chromatography–mass spectrometry [LCMS]), purification by preparative high-performance liquid chromatography (HPLC) (0.1% trifluoroacetic acid [TFA] in H_2_O/CH_3_CN, 40% to 45% CH_3_CN) afforded intermediate 1. Ethylvanillin (CAS 121-32-4, 8.5 g, 51.2 mmol, 1.0 equiv) and Meldrum’s acid (CAS 2033-24-1, 7.4 g, 51.2 mmol, 1.0 equiv) in MeOH were stirred at 25 °C for 16 h. After completion (LCMS), concentration and column chromatography (ethyl acetate/petroleum ether = 15:85) gave intermediate 2 as a yellow solid. Intermediate 2 (14.0 g, 47.9 mmol, 0.8 equiv), 2-cyanoethanethioamide (CAS 7357-70-2, 6.0 g, 59.9 mmol, 1.0 equiv), and *N*-methylmorpholine (CAS 109-02-4, 6.0 g, 59.9 mmol, 1.0 equiv) in EtOH (100 ml) were stirred at 25 °C for 14 h, and the resulting precipitate was collected by filtration to give intermediate 3 as a pale yellow solid. Intermediate 1 (2.0 g, 3.4 mmol) was treated with TFA/dichloromethane (DCM) (1:1, 20 ml) at 25 °C for 16 h, and the solvent was removed under reduced pressure. Lyophilization afforded crude intermediate 4 as a yellow oil. To a solution of intermediate 4 (1.3 g, 3.2 mmol, 1.0 equiv) and triethylamine (654 mg, 6.4 mmol, 2.0 equiv) in DCM (20 ml) at 0 °C was added chloroacetyl chloride (366 mg, 3.2 mmol, 1.0 equiv). After stirring for 2 h, the reaction mixture was purified by preparative HPLC to afford intermediate 5 as a brown oil. Intermediate 5 (1.3 g, 1.26 mmol, 1.0 equiv) and intermediate 3 (680 mg, 1.38 mmol, 1.1 equiv) in EtOH/H_2_O (8:2, 10 ml) were stirred at 90 °C for 2 h. Purification by preparative HPLC afforded intermediate 6 as a brown oil. Finally, intermediate 6 (180 mg, 0.20 mmol, 1.0 equiv), (R,S,S)-VH032 (CAS 2230826-33-0, 90 mg, 0.20 mmol, 1.0 equiv), and *N*,*N*-diisopropylethylamine (DIPEA) (54 mg, 0.40 mmol, 2.0 equiv) were dissolved in DMF (3 ml), followed by the addition of *O*-(7-azabenzotriazol-1-yl)-*N*,*N*,*N*′,*N*′-tetramethyluronium hexafluorophosphate (HATU; 95 mg, 0.24 mmol, 1.2 equiv). The mixture was stirred at 25 °C for 16 h. Intermediate 5 (1.3 g, 1.26 mmol, 1.0 equiv) and intermediate 3 (680 mg, 1.38 mmol, 1.1 equiv) in EtOH/H_2_O (8:2, 10 ml) were stirred at 90 °C for 2 h. Purification by preparative HPLC afforded intermediate 6 as a brown oil. Finally, intermediate 6 (180 mg, 0.20 mmol, 1.0 equiv), (R,S,S)-VH032 (CAS 2230826-33-0, 90 mg, 0.20 mmol, 1.0 equiv), and DIPEA (54 mg, 0.40 mmol, 2.0 equiv) were dissolved in DMF (3 ml), followed by the addition of HATU (95 mg, 0.24 mmol, 1.2 equiv). The mixture was stirred at 25 °C for 16 h. After completion (LCMS), preparative HPLC purification furnished NP-1 as a yellow solid.

### Public data sources

Gene correlation analysis was performed using RNA-seq data from TCGA-COAD and TCGA-READ cohorts of The Cancer Genome Atlas (TCGA) obtained from the Genomic Data Commons Data Portal (https://portal.gdc.cancer.gov/). Summaries of NSUN2 genomic alterations across cancers and its somatic mutation frequency along the protein were obtained from cBioPortal for Cancer Genomics (https://www.cbioportal.org/). Survival analyses of immunotherapy-treated patients were performed using the datasets melanoma-GSE91061, GBM-PRJNA482620, melanoma-PRJEB23709, and melanoma-Nathanson_2017 from the Tumor Immunotherapy Gene Expression Resource (TIGER) database (http://tiger.canceromics.org) or the Kaplan-Meier Plotter database (https://kmplot.com/analysis/). The expression profile of the NSUN2 gene across diverse human normal organs and tissues was analyzed and visualized using the Genotype-Tissue Expression Portal (https://www.gtexportal.org/home/). The E-MTAB-8107 dataset was downloaded from the European Molecular Biology Laboratory–European Bioinformatics Institute ArrayExpress database (https://www.ebi.ac.uk/), while the GSE166555 dataset was obtained from the National Center for Biotechnology Information Gene Expression Omnibus database (https://www.ncbi.nlm.nih.gov/geo/). The E-MTAB-8107 and GSE166555 single-cell RNA-seq datasets were analyzed using the Seurat package (v5.4.0) in R (v4.2.3). Following QC, normalization, and principal component analysis, cells were clustered and visualized by uniform manifold approximation and projection. Cell identities were annotated using SingleR (v2.6.1). Gene expression patterns across different cell populations were displayed using violin plots generated with Seurat package.

### Statistical analysis

Statistical analyses were performed using the GraphPad Prism 9 software. Spearman correlation analysis was used to determine the association between groups, whereas group comparisons were evaluated using 2-way analysis of variance or 2-tailed paired/unpaired *t* tests, as appropriate. Survival data were analyzed using Kaplan–Meier curves with log-rank tests. Statistical significance was defined as *P* < 0.05.

## Results

### NSUN2 functioned as a driver of cancer immune evasion independent of the catalytic activity

**Fig. 1.**
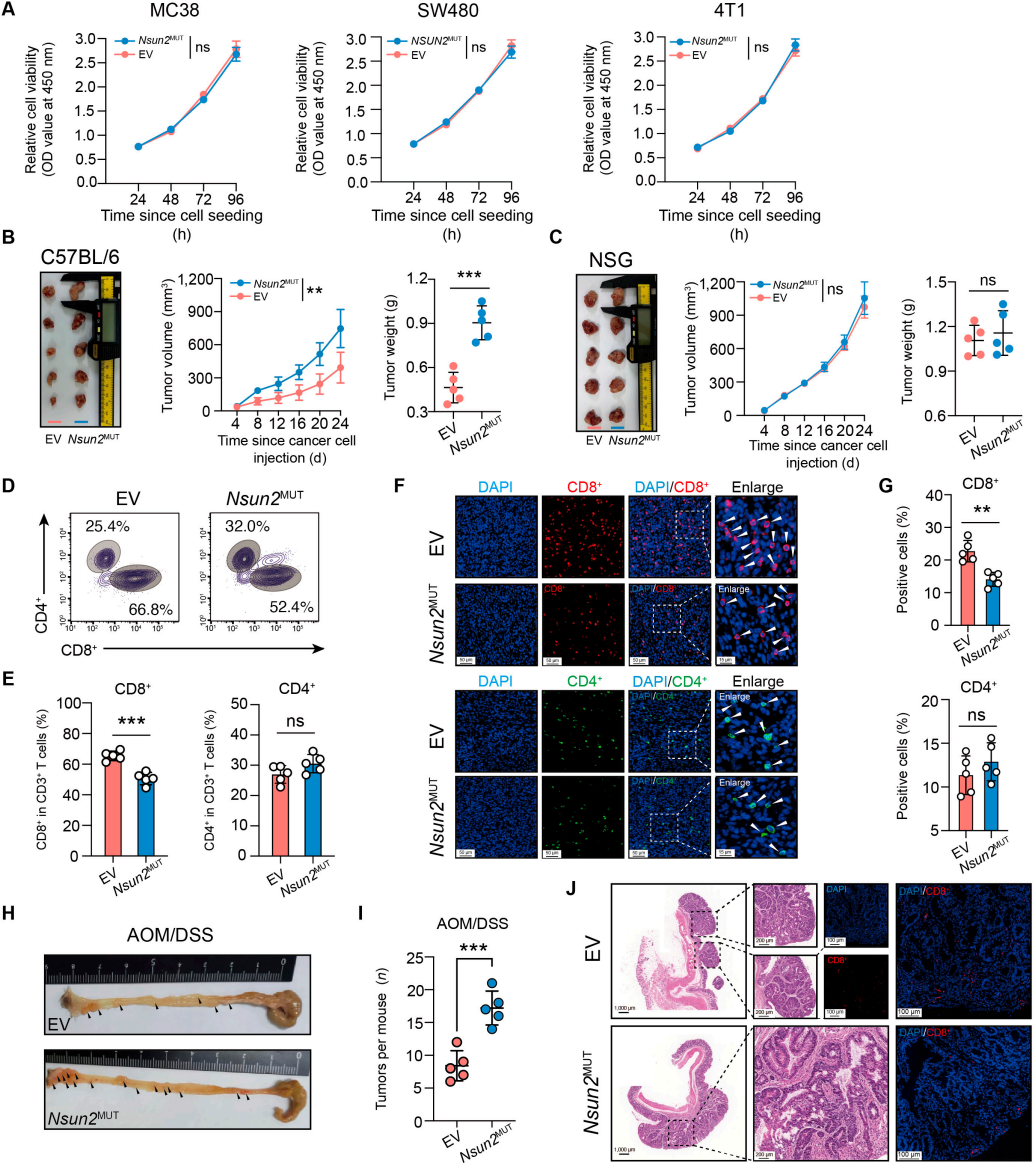
NSUN2 functioned as a driver of cancer immune evasion independent of the catalytic activity. (A) CCK-8 assay comparing EV and *Nsun2*^MUT^ MC38, SW480, and 4T1 cells. *n* = 3 biologically independent samples. (B) Representative images of excised tumors from each group. Tumor growth curves and endpoint tumor weights of mice bearing EV or *Nsun2*^MUT^ MC38 tumors in C57BL/6J (immunocompetent) mice (*n* = 5 per group). (C) Representative images of excised tumors from each group. Tumor growth curves and endpoint tumor weights of mice bearing EV or *Nsun2*^MUT^ MC38 tumors in NSG (immunodeficient) mice (*n* = 5 per group). (D) Representative plots and percentages of tumor-infiltrating CD8^+^ T cells and CD4^+^ T cells in EV and *Nsun2*^MUT^ MC38 tumors from immunocompetent C57BL/6J mice; *n* = 5 mice per group. (E) Statistical analysis of the percentages of CD4^+^ and CD8^+^ T cells from EV and *Nsun2*^MUT^ MC38 tumors using flow cytometry. (F) Representative IF staining images of CD4^+^ and CD8^+^ T cells from EV and *Nsun2*^MUT^ MC38 tumors (white arrowheads indicate CD4^+^ and CD8^+^ T cells, respectively); *n* = 5 mice per group. Three fields from one independent tumor from each mouse were analyzed. (G) Statistical analysis of the percentage of CD8^+^ and CD4^+^ T cells from EV and *Nsun2*^MUT^ MC38 tumors using IF. (H to J) Representative images of the tumor-bearing colon and rectum with arrows indicating tumors (H), tumor number per mouse (I), and representative H&E staining and IF staining images of CD4^+^ and CD8^+^ T cells in colonic tissues (J) from EV and *Nsun2*^MUT^ mice at the end of the experiment. Results are presented as the mean ± SD. *P < 0.05; **P < 0.01; ***P < 0.001; ns, not significant, as determined by an unpaired *t* test. Abbreviations: CCK-8, Cell Counting Kit-8; EV, empty vector; H&E, hematoxylin and eosin; IF, immunofluorescence; MUT, mutant; NSG, NOD-*Prkdc*^scid^-*Il2rg*^null^ mice; Nsun2, NOP2/Sun RNA methyltransferase 2; SD, standard deviation.

The classical m^5^C catalytic function has been widely implicated in tumorigenesis, offering insights that may lead to innovative therapeutic strategies [12,15]. In a previous study, we established the canonical role of NSUN2 in promoting proliferation, invasion, and metastasis of CRC through the NSUN2/YBX1/m^5^C-ENO1 signaling axis [15]. According to public cancer genomics databases, NSUN2 mutations were detected in a subset of tumors, including some within the RNA methyltransferase domain (MTD, amino acids 66 to 430) (Fig. S1A and B); however, whether mutations in this region lead to noncanonical functions is still undetermined. Thus, in the present study, we explored the noncanonical functions of NSUN2 in tumorigenesis and tumor immunity. A methyltransferase-dead NSUN2 mutant (*Nsun2*^MUT^) was genetically overexpressed in MC38, 4T1, and SW480 cells, which did not significantly affect in vitro cell growth compared with that of the EV group (Fig. 1A and Fig. S1C and D). *Nsun2*^MUT^ and control cells were implanted subcutaneously into immunodeficient NSG and immunocompetent C57BL/6 mice. Compared with the control EV group, overexpression of *Nsun2*^MUT^ markedly promoted MC38 tumor growth and was associated with reduced survival in immunocompetent C57BL/6 mice, whereas no significant effect was detected in NSG mice (Fig. 1B and C and Fig. S1E). To further analyze these observations, *Nsun2*^MUT^-overexpressed 4T1 cells were implanted into NSG and BALB/c mice. In line with the findings in MC38 cells, *Nsun2*^MUT^ overexpression had no effect on tumor growth in immunodeficient NSG mice but significantly enhanced tumor progression and was associated with poorer prognosis in immunocompetent BALB/c mice (Fig. S1F and G). These findings indicated that tumor-intrinsic NSUN2 might function as a key immunosuppressive regulator, facilitating immune evasion independent of its canonical catalytic activity. The effect of methyltransferase-dead NSUN2 on the cancer immune landscape was further explored using flow cytometry. Flow cytometry and IF analysis of subcutaneous tumors from immunocompetent mice showed that the overexpression of *Nsun2*^MUT^ reduced the proportion of tumor-infiltrating CD8^+^ T cells, whereas the proportion of CD4^+^ T cells remained unchanged compared with that in the EV group (Fig. 1D to G). To assess the effect of the *Nsun2*^MUT^ overexpression on intestinal tumorigenesis in situ, mice were administered an intestinal epithelium-specific recombinant adenovirus encoding methyltransferase-dead *Nsun2*^MUT^ or EV, followed by the induction of CRC using the AOM/DSS protocol. Unexpectedly, *Nsun2*^MUT^ mice showed significantly reduced survival (Fig. S1H) and developed a greater number of larger CRCs (Fig. 1H and I and Fig. S1I) compared with the EV group. IF staining consistently revealed that *Nsun2*^MUT^ overexpression markedly diminished CD8^+^ T-cell infiltration (Fig. 1J). Together, these findings demonstrated that NSUN2 drives cancer immune evasion independent of its enzymatic activity.

### NSUN2 promoted TCA cycle reprogramming and chemokine production through a noncatalytic mechanism

**Fig. 2.**
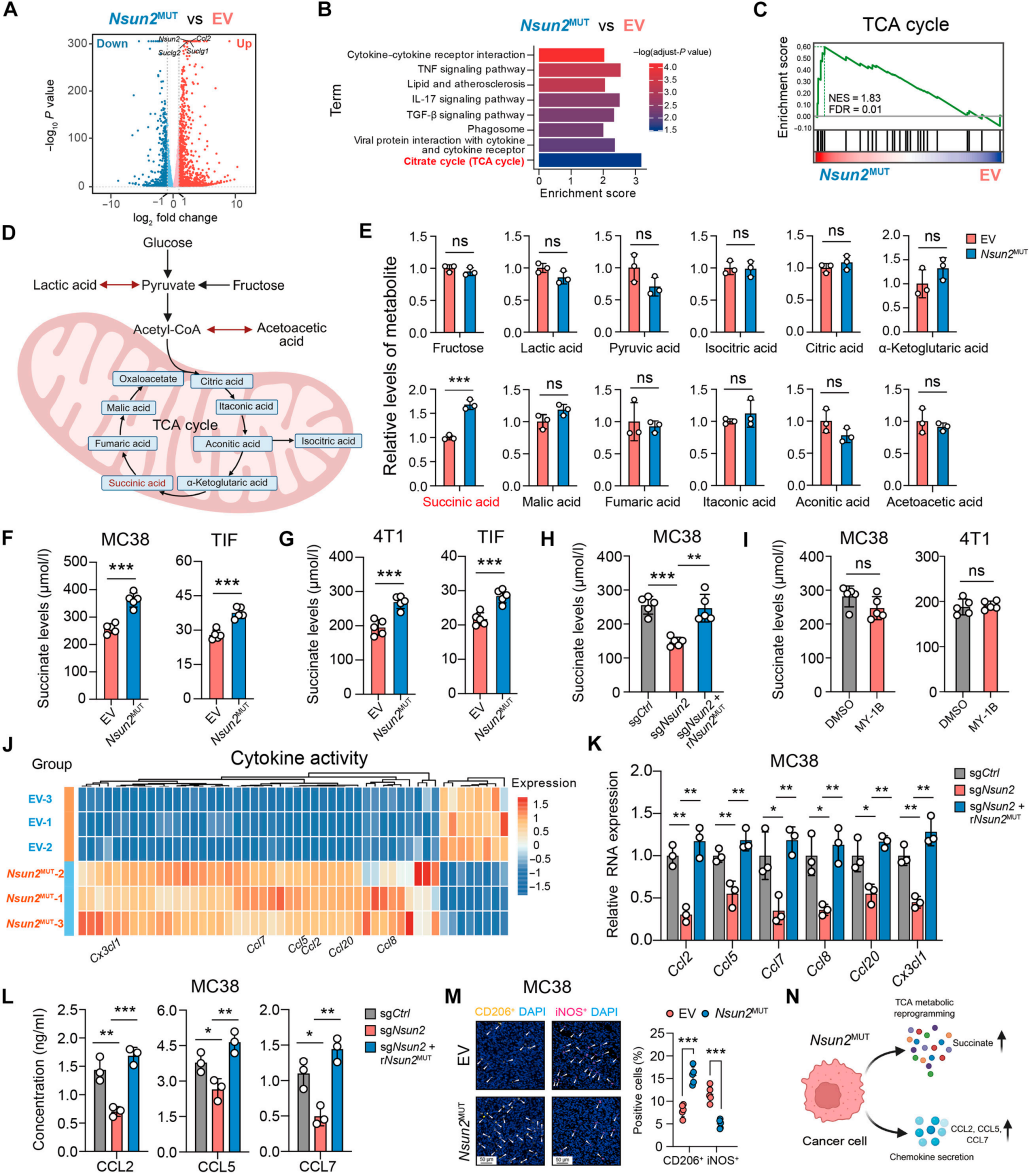
NSUN2 promoted TCA metabolic reprogramming and chemokine production independently of the catalytic activity. (A) Volcano plot showing DEGs from transcriptomic analyses of sorted mCherry^+^CD45^−^ tumor cells isolated from subcutaneous tumors of the EV and *Nsun2*^MUT^ groups. (B) KEGG pathway enrichment analysis of the DEGs in *Nsun2*^MUT^ compared to the EV group. (C) GSEA to assess specific enrichment of the TCA cycle from MC38 *Nsun2*^MUT^ and EV groups based on the WikiPathways dataset. (D) Schematic representation of the TCA cycle, showing major metabolites and key enzymes [42]. (E) Relative metabolite levels in EV and *Nsun2*^MUT^ tumors as determined by TCA cycle metabolite analysis. (F) Measurement of succinate levels in tumors and TIF from C57BL/6 tumor-bearing mice implanted with *Nsun2*^MUT^- or EV-derived MC38 cells, with 5 mice per group. (G) Measurement of succinate levels in tumors and TIF from BALB/c tumor-bearing mice implanted with *Nsun2*^MUT^- or EV-derived 4T1 cells, with 5 mice per group. (H) Determination of succinate levels in sg*Ctrl*, sg*Nsun2*, and sg*Nsun2* + *Nsun2*^MUT^ MC38 cells. (I) Determination of succinate levels in MC38 and 4T1 cells treated with DMSO or 5 μmol/l MY-1B. (J) Heatmap illustrating the differential transcriptional profiles of cytokine-activity-associated genes between *Nsun2*^MUT^ and EV groups (data from (A)). (K) RT-qPCR analysis of the expression levels of 6 genes from sg*Ctrl*, sg*Nsun2*, or sg*Nsun2* + r*Nsun2*^MUT^ MC38 cells. (L) ELISA analysis of the CCL2, CCL5, and CCL7 chemokine levels from sg*Ctrl*, sg*Nsun2*, or sg*Nsun2* + r*Nsun2*^MUT^ MC38 cells. (M) Representative IF staining images and statistical analysis of CD206^+^ and iNOS^+^ TAM cells from EV and *Nsun2*^MUT^ MC38 tumors; *n* = 5 mice per group. (N) Schematic illustration of catalytic-activity-independent roles of NSUN2 in driving TCA cycle reprogramming and up-regulating the expression of chemokines CCL2, CCL5, and CCL7 in CRC cells. Results are presented as mean ± SD. *P < 0.05; **P < 0.01; ***P < 0.001; ns, not significant, as determined by an unpaired *t* test. Abbreviations: CCL2/5/7, C-C motif chemokine ligand 2/5/7; CRC, colorectal cancer; DEGs, differentially expressed genes; DMSO, dimethyl sulfoxide; ELISA, enzyme-linked immunosorbent assay; EV, empty vector; GSEA, gene set enrichment analysis; IF, immunofluorescence; iNOS, inducible nitric oxide synthase; KEGG, Kyoto Encyclopedia of Genes and Genomes; MUT, mutant; NSUN2, NOP2/Sun RNA methyltransferase 2; RT-qPCR, reverse transcription quantitative polymerase chain reaction; SD, standard deviation; sg*Ctrl*, single-guide RNA control; TAM, tumor-associated macrophages; TCA, tricarboxylic acid cycle; TIF, tumor interstitial fluid.

To elucidate the mechanisms through which NSUN2 drives cancer immune evasion independent of its catalytic activity, sorted mCherry^+^CD45^−^ tumor cells isolated from the subcutaneous tumors of the EV and *Nsun2*^MUT^ groups were subjected to RNA-seq. A total of 1,611 DEGs were identified, including 1,086 up-regulated and 525 down-regulated genes, in *Nsun2*^MUT^ tumors compared with the EV group (Fig. 2A). KEGG analysis revealed that these DEGs were enriched in several immune-related pathways, such as cytokine–cytokine receptor interaction and IL-17 signaling pathway, among which the TCA cycle had the highest enrichment score (Fig. 2B). GSEA based on the WikiPathways database revealed robust enrichment of the TCA cycle in the *Nsun2*^MUT^ group (Fig. 2C). To determine whether *Nsun2*^MUT^-mediated transcriptional remodeling of the TCA cycle affects metabolic alterations, targeted metabolomics was performed to identify differential metabolites between the EV and *Nsun2*^MUT^ MC38 groups. The results revealed elevated levels of succinate in the *Nsun2*^MUT^ MC38 group compared with those in the EV group (Fig. 2D and E). The phenotype was further confirmed by higher succinate levels in subcutaneous tumors derived from *Nsun2*^MUT^ MC38 or 4T1 cells compared to those from the EV control group in immunocompetent mice, accompanied by the same upward trend in tumor interstitial fluid (Fig. 2F and G). Moreover, NSUN2 knockout led to a pronounced decrease in endogenous succinate levels compared to the sg*Ctrl* group, whereas re-expression of *Nsun2*^MUT^ effectively restored succinate levels in MC38 and 4T1 cancer cells (Fig. 2H and Fig. S2A and B). However, succinate levels did not differ significantly between cancer cells treated with DMSO and those treated with the NSUN2-targeting m^5^C methyltransferase inhibitor MY-1B (Fig. 2I), further supporting a noncanonical catalytic activity-independent function of NSUN2 in modulating TCA metabolic reprogramming. In addition, GSEA revealed a marked enrichment of cytokine activity, chemokine activity, and chemoattractant activity pathways in the *Nsun2*^MUT^ group (Fig. S2C). As depicted, macrophage-recruiting chemokines, including *Ccl2*, *Ccl5*, *Ccl7*, *Ccl8*, and *Cx3cl1*, were increased in *Nsun2*^MUT^ MC38 tumors (Fig. 2J). Consistent with this, RT-qPCR analysis confirmed that several chemokine genes, including *Ccl2*, *Ccl5*, *Ccl7*, *Ccl8*, and *Cx3cl1*, were markedly down-regulated in NSUN2-knockout MC38 and SW480 cells, whereas re-expression of *Nsun2*^MUT^ effectively restored their expression, and MY-1B treatment had no appreciable effect (Fig. 2K and Fig. S2D and E). ELISA further confirmed that chemokine levels were markedly reduced in NSUN2-knockout MC38, SW480, and 4T1 cells, whereas re-expression of *Nsun2*^MUT^ effectively restored their production, and MY-1B treatment had no detectable effect (Fig. 2L and Fig. S2F and G). IF staining analysis demonstrated significantly elevated levels of TAM2 cells (CD206^+^) in the TME of *Nsun2*^MUT^ MC38 tumors compared to the EV control group, whereas TAM1 cells (iNOS^+^) showed reduced infiltration (Fig. 2M). These findings indicated that NSUN2 orchestrates the metabolic reprogramming of the TCA cycle and fosters the development of immunosuppressive TAMs, independent of its catalytic activity (Fig. 2N).

### NSUN2 interacted with GATA3 driving SUCLG1/SUCLG2 and chemokine expression

**Fig. 3.**
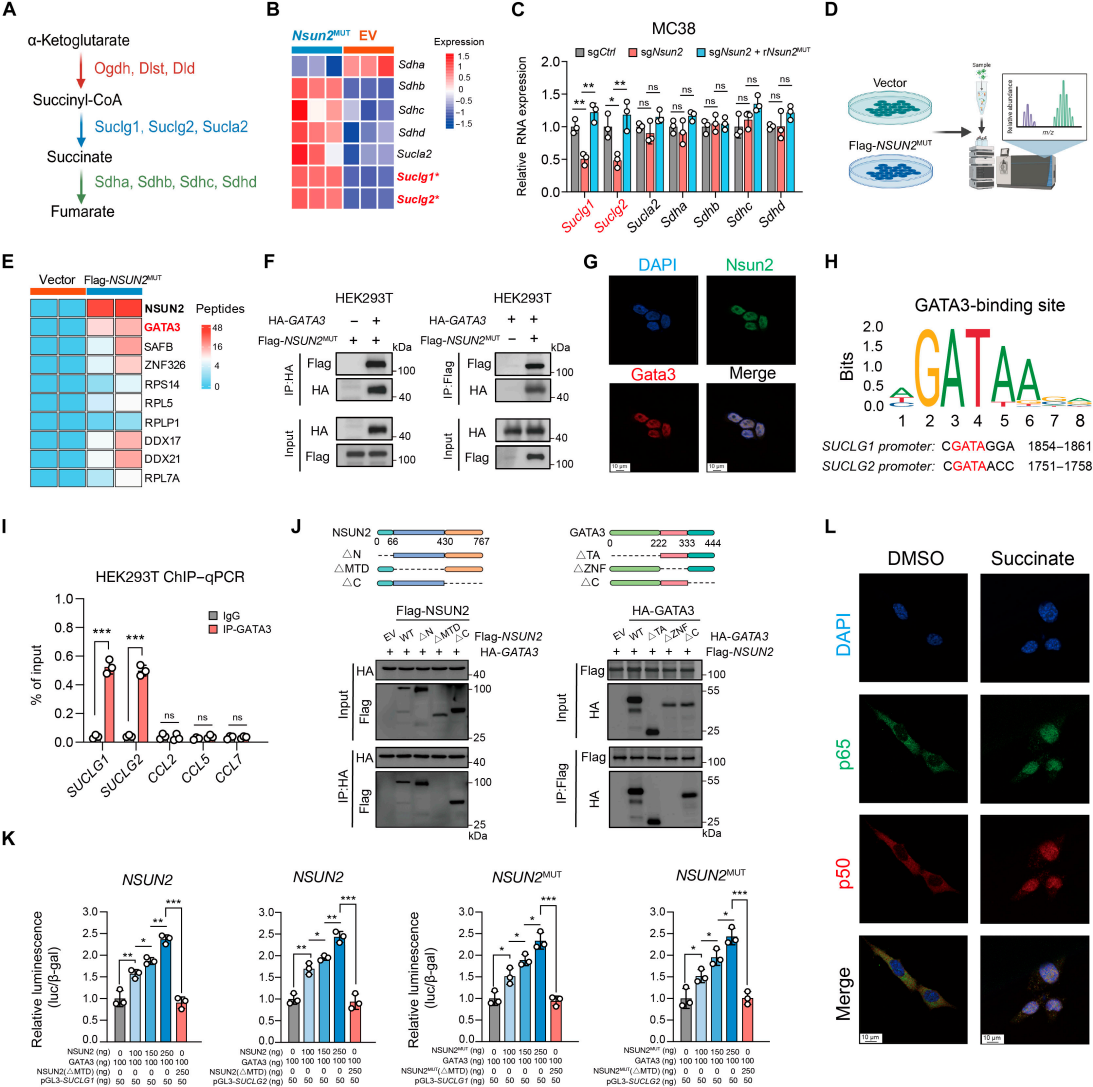
NSUN2 acted as a cofactor of GATA3 and promoted the expression of SUCLG1/SUCLG2 and chemokines. (A) Schematic representation of the critical regulatory proteins associated with succinate metabolism. (B) Heatmap showing the differential expression of succinate-related metabolic genes in sorted mCherry^+^CD45^−^ tumor cells isolated from subcutaneous tumors of the EV and *Nsun2*^MUT^ groups (data from Fig. 2A). (C) RT-qPCR analysis of the expression levels of the succinate-related genes in sg*Ctrl*, sg*Nsun2*, and sg*Nsun2* + *Nsun2*^MUT^-reconstituted MC38 cells; *n* = 3 samples for each group. (D) Schematic representation of anti-Flag antibody-mediated IP and MS analysis of Flag-*NSUN2*^MUT^ and EV group samples. (E) Heatmap depicting differentially abundant proteins identified through MS analysis in HEK293T cells expressing Flag-*NSUN2*^MUT^ compared with control EV groups (*n* = 2 per group). (F) WB analysis of whole-cell lysate and IP from HEK293T cells transfected with Flag-*NSUN2*^MUT^, HA-GATA3, or both in combination. IP was performed with anti-HA or anti-Flag antibodies, followed by WB with the indicated antibodies to detect the physical interaction between *NSUN2*^MUT^ and GATA3. (G) Representative IF staining images of NSUN2 and GATA3 in MC38 cells. (H) Predicted GATA3-binding sites on *SUCLG1* and *SUCLG2* promoters. (I) ChIP–qPCR analysis of GATA3 binding to the promoters of *SUCLG1*, *SUCLG2*, *CCL2*, *CCL5*, and *CCL7* promoters in HEK293T cells; *n* = 3 samples for each group. (J) Schematic representation of truncated forms of NSUN2 and GATA3. WB analysis of WCL and anti-Flag or anti-HA IP from HEK293T cells transfected with the MTD, TA, and ZNF constructs. (K) Luciferase reporter analysis of the transcriptional activation of *SUCLG1* and *SUCLG2* mediated by *NSUN2*, its ΔMTD variants, *NSUN2*^MUT^, or the corresponding ΔMTD variants. (L) Indirect IF of p65, p50, and DAPI in MC38 cells treated with succinate or DMSO. Results are presented as mean ± SD. *P < 0.05; **P < 0.01; ***P < 0.001; ns, not significant through an unpaired *t* test. Abbreviations: ChIP–qPCR, chromatin immunoprecipitation–quantitative polymerase chain reaction; DAPI, 4′,6-diamidino-2-phenylindole; DMSO, dimethyl sulfoxide; EV, empty vector; GATA3, GATA-binding protein 3; IF, immunofluorescence; IP, immunoprecipitation; MS, mass spectrometry; MTD, methyltransferase domain; MUT, mutant; NSUN2, NOP2/Sun RNA methyltransferase 2; RT-qPCR, reverse transcription quantitative polymerase chain reaction; SD, standard deviation; sg*Ctrl*, single-guide RNA control; SUCLG1, succinate-CoA ligase GDP/ADP-forming subunit α; SUCLG2, succinate-CoA ligase GDP/ADP-forming subunit β; TA, transactivation domain; WB, western blotting; ZNF, zinc finger domain.

Our findings challenge the established paradigm that NSUN2 functions exclusively as an RNA methylation enzyme. To elucidate the molecular mechanisms through which NSUN2 regulates the expression of TCA cycle metabolic genes, we investigated whether these genes were direct transcriptional targets of NSUN2 (Fig. 3A). The key enzymes upstream and downstream of succinate involved in the TCA cycle were further investigated; transcriptomic data showed that *Suclg1* and *Suclg2* were up-regulated in the *Nsun2*^MUT^ group relative to those in the EV group (Fig. 3B), while RT-qPCR analysis verified that their expression was significantly reduced in the sg*Nsun2* group in MC38 and SW480 cells, with restored expression following *Nsun2*^MUT^ reconstitution, whereas no obvious expression changes were observed for other succinate-related genes (Fig. 3C and Fig. S3A). Consistent with the previous findings, no difference in *Suclg1* and *Suclg2* expression was observed upon treatment with the NSUN2 m^5^C methyltransferase inhibitor MY-1B (Fig. S3B). Given that NSUN2 recognized RNA and exerted its functions through direct RNA binding based on published evidence [15], a RIP–qPCR assay was performed to examine its potential direct interaction with *SUCLG1* and *SUCLG2* transcripts, and the result revealed that NSUN2 failed to directly bind to *SUCLG1* or *SUCLG2* transcripts in contrast to its strong binding to *ENO1* mRNA (Fig. S3C), a well-known NSUN2-binding transcript used as the positive control [15]. Therefore, we hypothesized that NSUN2 may interact with transcription factors to promote the transcription of *SUCLG1* and *SUCLG2* via a noncanonical mechanism. IP–MS analysis was subsequently conducted to identify NSUN2-interacting transcription factors that may cooperatively promote the expression of *Suclg1* and *Suclg2* (Fig. 3D). This analysis revealed that several of the evident candidate transcription factors, including GATA3, were significantly enriched in *NSUN2*^MUT^-overexpressing cells (Fig. 3E). Combined analysis of the public datasets of TCGA-COAD and TCGA-READ cohorts further demonstrated that the expression of *Suclg1* and *Suclg2* was significantly inversely correlated with that of the transcription factor GATA3 (Fig. S3D). Co-IP assays revealed strong interactions of both exogenous and endogenous NSUN2 with GATA3 (Fig. 3F and Fig. S3E). Furthermore, we examined whether GATA3 selectively interacts with NSUN2 or associates with other NSUN family members and found that GATA3 exhibited weaker interactions with NSUN5 and NSUN6, likely attributable to its structural similarity (Fig. S3F). Co-IP assays were performed using cell lysates pretreated with DNase I to degrade DNA. Importantly, GATA3–NSUN2 interaction remained detectable after DNase I treatment, indicating that the interaction occurs independently of chromatin (Fig. S3G). Consistent with this, Nsun2 and Gata3 displayed strong colocalization in MC38 cells (Fig. 3G). Following treatment with the GATA3 inhibitor pyrrothiogatain, the levels of *SUCLG1* and *SUCLG2* increased, whereas those of *CCL2*, *CCL5*, and *CCL7* remained unaltered in SW480 cells (Fig. S3H). To determine whether NSUN2 regulates target genes by binding to GATA3, we performed ChIP–qPCR and determined GATA3 occupancy on the promoters of the TCA cycle metabolic genes (Fig. 3H). ChIP–qPCR analysis showed that *SUCLG1* and *SUCLG2* were enriched in the GATA3 group compared with those in the IgG control group, whereas no significant enrichment was observed for *CCL2*, *CCL5*, or *CCL7* (Fig. 3I). In parallel, ChIP–qPCR analysis was performed to determine whether NSUN2 binds to GATA3 target promoters; however, no enrichment of NSUN2 was detected at the promoters of the GATA3 target genes identified in this study (*SUCLG1* and *SUCLG2*) or at the promoter of the reported downstream target of GATA3, *FOXA1* (Fig. S3I) [23]. Furthermore, knockout of *NSUN2* markedly increased enrichment at the promoters of *SUCLG1* and *SUCLG2*, whereas this effect was reduced upon reconstitution with *NSUN2*^MUT^ (Fig. S3J and K). The association with GATA3 was mediated by the MTD of NSUN2 (Fig. 3J). Conversely, this interaction was mediated by the zinc finger domain of GATA3 (Fig. 3J). Collectively, these results provide strong evidence for the identification of NSUN2 as a GATA3-binding partner, which relieves the repression of *SUCLG1* and *SUCLG2* exerted by GATA3. *Gata3* knockout and overexpression cell models were generated, and RT-qPCR analysis revealed that *Suclg1* and *Suclg2* expression remained unchanged in the sg*Ctrl*, sg*Nsun2*, and sg*Nsun2* + *Nsun2*^MUT^-reconstituted *Gata3*-deficient cells, whereas in *Nsun2*-deficient cells, *Gata3* knockout markedly increased the expression of *Suclg1* and *Suclg2* (Fig. S3L and M). In *Gata3*-overexpression cells, RT-qPCR analysis indicated that the expression levels of *Suclg1* and *Suclg2* were significantly reduced compared with those in the EV group across all experimental groups, including sg*Ctrl*, sg*Nsun2*, and sg*Nsun2* + r*Nsun2*^MUT^-reconstituted groups; compared with the sg*Ctrl* group, *Nsun2* knockout further exacerbated this repressive effect, whereas reconstitution of r*Nsun2*^MUT^ significantly restored *Suclg1* and *Suclg2* expression levels in MC38 cells with *Gata3* overexpression (Fig. S3N). Both *NSUN2* and *NSUN2*^MUT^ facilitated the ability of their cofactors to relieve GATA3-mediated repression of pGL3-*SUCLG1* and pGL3-*SUCLG2* transcription, whereas *NSUN5* and *NSUN6* failed to exert this effect in luciferase reporter assays (Fig. 3K and Fig. S3O and P). Moreover, WT *NSUN2* or *NSUN2*^MUT^, but not *NSUN2* (ΔMTD), activated the transcription of pGL3-*SUCLG1* and pGL3-*SUCLG2* (Fig. 3K). Collectively, these results support a model in which NSUN2 functions as a selective activating cofactor that antagonizes the transcriptional repressor activity of GATA3, in contrast to NSUN5 and NSUN6, thereby promoting the transcription of *SUCLG1* and *SUCLG2* and increasing succinate levels. However, our above data confirmed that NSUN2 up-regulated CCL2, CCL5, and CCL7 through a GATA3-independent pathway, and the relevant mechanism is yet to be clarified. Since succinate regulated NF-κB signaling and subsequent expression of macrophage-recruiting chemokines by driving nuclear translocation of the p65–p50 heterodimer [24], we therefore investigated this regulatory mechanism resulted from NSUN2-mediated succinate accumulation in CRC cells. Notably, succinate administration increased the nuclear levels of p65 and p50 in MC38 cells (Fig. 3L and Fig. S3Q). In line with this, nuclear p65 and p50 levels were reduced in sg*Nsun2* cells but were restored upon *Nsun2*^MUT^ reconstitution (Fig. S3R), indicating a role for NSUN2 in regulating NF-κB activation. In contrast, RelB expression, a key component of the noncanonical NF-κB pathway [24], remained unchanged following succinate treatment, in sg*Nsun2* cells, or upon *Nsun2*^MUT^ reconstitution (Fig. S3S and T), suggesting that the effect is specific to the canonical pathway. In summary, these findings reveal a molecular route through which NSUN2 orchestrates metabolic reprogramming of the TCA cycle and canonical NF-κB activation, bypassing its enzymatic role.

### NSUN2-mediated elevation of succinate orchestrated TAM reprogramming and facilitated immune evasion

**Fig. 4.**
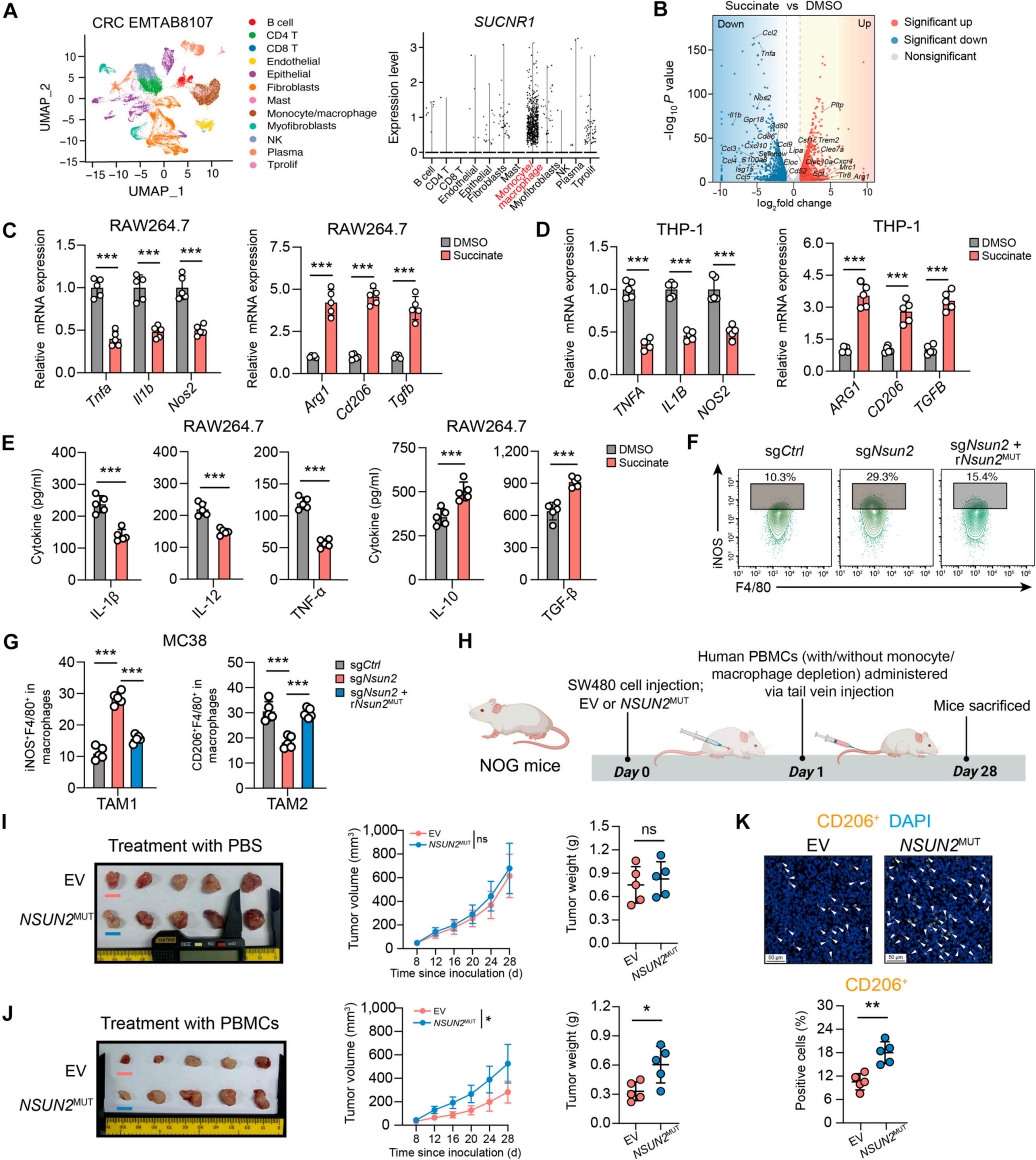
NSUN2-mediated elevation of succinate orchestrated TAM reprogramming and facilitated immune evasion. (A) Analysis of SUCNR1 expression across different immune cell types in CRC tissues using the E-MTAB-8107 database. (B) Volcano plot showing DEGs from transcriptomic analyses of RAW264.7 cells treated with 100 μmol/l succinate versus DMSO. (C) RT-qPCR analysis of the expression levels of TAM1-associated genes (*Tnfa*, *Il1b*, and *Nos2*) or TAM2-associated genes (*Arg1*, *Cd206*, *Tgfb*) in RAW264.7 cells treated with succinate compared to the DMSO group. (D) RT-qPCR analysis of the expression levels of TAM1-associated genes (*TNFA*, *IL1B*, and *NOS2*) or TAM2-associated genes (*ARG1*, *CD206*, and *TGFB*) in THP-1 cells treated with succinate compared to those of the DMSO groups. (E) ELISA analysis of the IL-1β, IL-12, TNF-α, IL-10, and TGF-β cytokine levels in RAW264.7 cells treated with DMSO or succinate. (F) Representative plots and percentages of iNOS^+^F4/80^+^ cells in co-culture with sg*Ctrl*, sg*Nsun2*, and r*Nsun2*^MUT^ MC38 cells with RAW264.7 cells. (G) Statistical analysis of iNOS^+^F4/80^+^ or CD206^+^F4/80^+^ cells from co-culture with sg*Ctrl*, sg*Nsun2*, and r*Nsun2*^MUT^ MC38 cells with RAW264.7 cells using FACS; *n* = 5 per group. (H) Schematic representation of the in vivo experimental regimen in NOG mice. (I) Representative tumor photographs, growth curves, and tumor weights of NOG mice bearing EV or *NSUN2*^MUT^ SW480 cells treated with PBS (*n* = 5 mice per group). (J) Representative tumor images, tumor growth curves, and final tumor weights in NOG mice implanted with EV or *NSUN2*^MUT^ SW480 cells and treated with PBMCs (*n* = 5 per group). (K) Representative IF staining images and statistical analysis of CD206^+^ TAMs from EV and *NSUN2*^MUT^ SW480 tumors treated with PBMCs; *n* = 5 mice per group. Three fields from one independent tumor of each mouse were analyzed. Results are presented as mean ± SD. *P < 0.05; **P < 0.01; ***P < 0.001; ns, not significant through unpaired *t* test. Abbreviations: ARG1, arginase 1; CRC, colorectal cancer; DEGs, differentially expressed genes; DMSO, dimethyl sulfoxide; ELISA, enzyme-linked immunosorbent assay; EV, empty vector; FACS, fluorescence-activated cell sorting; IF, immunofluorescence; IL-1β, interleukin 1 beta; IL-10, interleukin 10; IL-12, interleukin 12; iNOS, inducible nitric oxide synthase; MUT, mutant; NOG, NOD.Cg-*Prkdc^scid^Il2rg^tm1Sug^*/ShiJic; NSUN2, NOP2/Sun RNA methyltransferase 2; PBS, phosphate-buffered saline; PBMCs, peripheral blood mononuclear cells; RT-qPCR, reverse transcription quantitative polymerase chain reaction; SD, standard deviation; sg*Ctrl*, single-guide RNA control; SUCNR1, succinate receptor 1; TAM, tumor-associated macrophage; TGF-β, transforming growth factor beta; TNF-α, tumor necrosis factor alpha.

The role of NSUN2 in reprogramming TCA metabolism independent of its catalytic activity and in elevating succinate levels has been investigated above; however, the precise mechanism by which these elevated succinate levels induce tumor immune evasion remains unknown. Given that succinate is primarily transported via SUCNR1 [25], single-cell analysis revealed that SUCNR1 expression was markedly higher in macrophages than in other immune cell populations (Fig. 4A and Fig. S4A). Thus, succinate was added to the culture medium of mouse RAW264.7 macrophage cells, which were subjected to RNA-seq. In succinate-treated cells, the expression of macrophage TAM2 polarization-related genes, including *Arg1*, *Mrc1* (*Cd206*), and *Csf1r*, was significantly up-regulated, whereas TAM1-associated genes, such as *Tnfa*, *Nos2*, *Cd86*, and *Il1b*, were markedly down-regulated (Fig. 4B). In validation experiments, succinate treatment reduced the expression of TAM1-associated genes (*Tnfa*, *Il1b*, and *Nos2*) and increased the RNA expression of TAM2-associated genes (*Arg1*, *Cd206*, and *Tgfb*) in RAW264.7 cells (Fig. 4C). The same trend was consistently observed in human macrophage THP-1 cells and BMDMs (Fig. 4D and Fig. S4B). ELISA further confirmed that the succinate treatment significantly reduced the secretion of IL-1β, IL-12, and TNF-α while increasing the levels of IL-10 and TGF-β in RAW264.7 or BMDMs compared with DMSO controls (Fig. 4E and Fig. S4C). In addition, flow cytometry of sg*Ctrl*, sg*Nsun2*, and r*Nsun2*^MUT^ MC38 or 4T1 cancer cells co-cultured with BMDMs revealed a marked decrease in the abundance of TAM2 macrophages in the sg*Nsun2* group compared with that in the control group, which was restored in the sg*Nsun2* + r*Nsun2*^MUT^ group, accompanied by an opposite trend in TAM1 macrophages (Fig. 4F and G and Fig. S4D). To evaluate the contribution of macrophages to cancer growth inhibition triggered by the noncatalytic function of NSUN2, tumor growth was evaluated for EV or *NSUN2*^MUT^ using a xenograft model in NOG mice with or without transfusion of isolated human PBMCs (with or without monocyte/macrophage depletion) to create a humanized immune environment (Fig. 4H). Under these conditions, *NSUN2*^MUT^ did not alter the tumor growth or weight in NOG mice (Fig. 4I). In contrast, in the group of mice receiving PBMCs, *NSUN2*^MUT^ significantly increased tumor growth and weight in NOG mice compared with those in the EV group (Fig. 4J). To determine the role of monocytes/macrophages in antitumor immunity, we assessed tumor progression in NOG mice engrafted with PBMCs depleted of these cells and found that tumor growth and final tumor weights were not significantly altered compared with those in the EV control group (Fig. S4E). Furthermore, overexpression of *NSUN2*^MUT^ increased the expression of the TAM2 macrophage marker CD206 in tumors from NOG mice engrafted with PBMCs (Fig. 4K), indicating a shift toward an immunosuppressive microenvironment associated with the moonlighting function of NSUN2. Thus, these findings indicate that macrophages are indispensable for the tumor-promoting effects of NSUN2 in vivo and point to an SUCLG1/SUCLG2–succinate–TAM polarization–T cell suppression axis as the central mechanism through which NSUN2 facilitates tumor immune evasion independent of its classical enzymatic activity.

### Succinate promoted TAM reprogramming and immune evasion via the cGAS–STING pathway

**Fig. 5.**
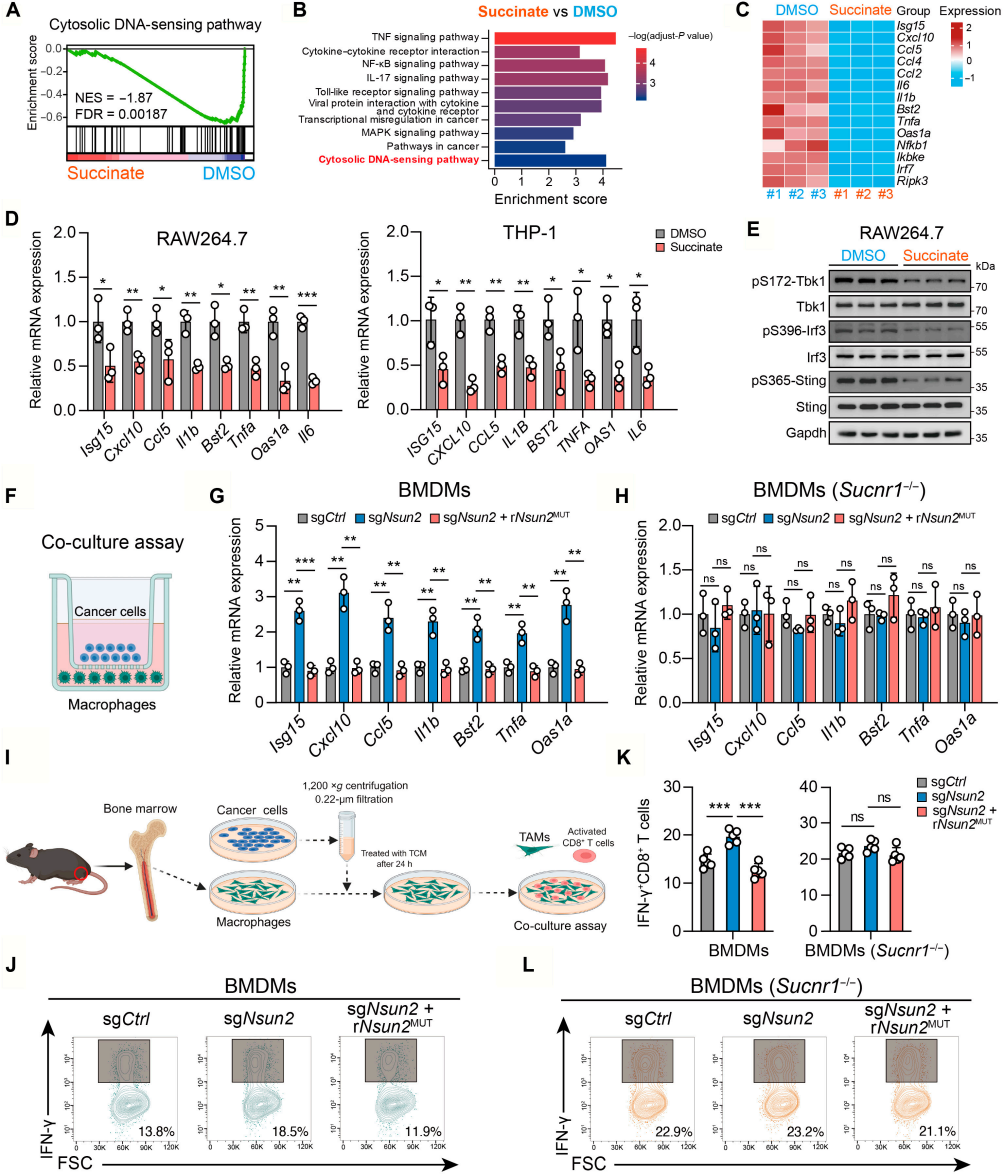
Succinate orchestrates TAM reprogramming and immune evasion via cGAS–STING pathway. (A) GSEA to assess specific enrichment of the cytosolic DNA-sensing pathway in RAW264.7 cells treated with succinate or DMSO. (B) KEGG pathway enrichment of the DEGs in RAW264.7 cells treated with succinate compared to those treated with DMSO. (C) Heatmap showing differential transcriptomic expression of cytosolic DNA-sensing pathway genes in RAW264.7 cells treated with succinate compared to those treated with DMSO. (D) RT-qPCR analysis of the cytosolic DNA-sensing pathway gene expression level in RAW264.7 or THP-1 cells treated with succinate compared to those treated with DMSO. (E) WB analysis of the indicated protein levels in RAW264.7 cells treated with DMSO or succinate. (F) Schematic representation of the co-culture assay of cancer cells with macrophages. (G) RT-qPCR analysis of the expression levels of interferon-stimulated genes in BMDMs co-cultured with sg*Ctrl*, sg*Nsun2*, or sg*Nsun2* + r*Nsun2*^MUT^ MC38 cells. (H) RT-qPCR analysis of the expression levels of interferon-stimulated genes from *Sucnr1* knockout BMDMs co-cultured with sg*Ctrl*, sg*Nsun2*, or sg*Nsun2* + r*Nsun2*^MUT^ MC38 cells. (I) Schematic representation of the in vitro co-culture assay of CD8^+^ T cells with BMDMs in the presence of tumor-conditioned medium from different cancer cells. (J) Representative plots and percentages of IFN-γ^+^ CD8^+^ T cells from co-culture with sg*Ctrl*, sg*Nsun2*, and sg*Nsun2* + r*Nsun2*^MUT^ MC38 cells with BMDMs. (K) Statistical analysis of IFN-γ^+^ CD8^+^ T cells in (J) from FACS, *n* = 5 per group. (L) Representative plots and percentages of IFN-γ^+^CD8^+^ T cells from co-culture with sg*Ctrl*, sg*Nsun2*, and r*Nsun2*^MUT^ MC38 cells with *Sucnr1*^−/−^ BMDM cells. Results are presented as mean ± SD. *P < 0.05; **P < 0.01; ***P < 0.001; ns, not significant through an unpaired *t* test. Abbreviations: BMDMs, bone-marrow-derived macrophages; cGAS, cyclic GMP-AMP synthase; DEGs, differentially expressed genes; DMSO, dimethyl sulfoxide; FACS, fluorescence-activated cell sorting; GSEA, gene set enrichment analysis; IFN-γ, interferon gamma; KEGG, Kyoto Encyclopedia of Genes and Genomes; MUT, mutant; NSUN2, NOP2/Sun RNA methyltransferase 2; RT-qPCR, reverse transcription quantitative polymerase chain reaction; SD, standard deviation; sg*Ctrl*, single-guide RNA control; STING, stimulator of interferon genes; SUCNR1, succinate receptor 1; TAM, tumor-associated macrophages; WB, western blot.

To elucidate the molecular mechanisms through which succinate drives TAM reprogramming, RNA-seq-based bioinformatic analysis was conducted, which revealed DEGs enriched in the cytosolic DNA-sensing pathway in succinate-treated RAW264.7 cells through GSEA (Fig. 5A). KEGG analysis of the DEGs also revealed that the majority of the enriched pathways were related to innate immune responses, including the cytosolic DNA-sensing pathway, toll-like receptor, and NF-κB signaling pathway (Fig. 5B). The cGAS–STING pathway serves as the central axis of innate immune activation triggered by cytosolic DNA and is particularly activated by cytosolic mtDNA released during cellular stress [26]. Accordingly, heatmap analysis showed that 14 genes associated with the cGAS–STING signaling pathway were significantly down-regulated in succinate-treated RAW264.7 cells (Fig. 5C). TAMs promote antitumor immune responses through activation of the cGAS–STING signaling pathway [27]. In line with these findings, RT-qPCR confirmed the significant down-regulation of downstream cytokine genes in the cGAS–STING signaling pathway, including *Isg15*, *Cxcl10*, *Ccl5*, *Il1b*, *Bst2*, *Tnfa*, *Oas1a*, and *Il6*, in both mouse and human macrophages treated with succinate compared with that in DMSO-treated controls (Fig. 5D and Fig. S5A). To investigate whether succinate regulates the cGAS–STING signaling pathway, we analyzed the phosphorylation levels of Tbk1 at S172 (pS172-Tbk1), Irf3 at S396 (pS396-Irf3), and Sting at S366/S365 (pS366/pS365-Sting) in macrophages. Succinate treatment reduced pS172-Tbk1, pS396-Irf3, and pS365/366-Sting levels in RAW264.7 cells, with similar decreases observed in THP-1 cells (Fig. 5E and Fig. S5B). To elucidate the mechanism by which succinate suppresses the cGAS–STING pathway, we first examined mtDNA expression levels and found that succinate treatment did not reduce its abundance (Fig. S5C). Succinate has been suggested to affect cGAS activity in cancer cells, prompting an investigation into whether a similar mechanism operates in immune macrophages [26]. Consistently, luciferase reporter assays revealed that the transcriptional activity of the cGAS promoter was suppressed in succinate-treated cells (Fig. S5D). Moreover, RT-qPCR analysis demonstrated that succinate treatment decreased *cGAS* expression in both RAW264.7 and THP-1 cells (Fig. S5E), indicating that succinate suppresses the cGAS–STING signaling cascade through modulation of cGAS expression. A co-culture system was used to investigate how NSUN2-mediated succinate elevation modulates the cGAS–STING signaling pathway in macrophages, thereby attenuating antitumor immune responses (Fig. 5F). Co-culture of sg*Nsun2* MC38 cancer cells with BMDMs and RAW264.7 cells led to a marked up-regulation of downstream interferon-stimulated genes (ISGs), including *Isg15*, *Cxcl10*, *Ccl5*, *Il1b*, *Bst2*, *Tnfa*, and *Oas1a*, relative to those in co-culture with sg*Ctrl* MC38 cells, whereas reconstitution of *Nsun2*^MUT^ reversed the increased expression of these genes (Fig. 5G and Fig. S5F). However, co-culturing RAW264.7 cells with the GATA3 inhibitor pyrrothiogatain-treated MC38 cancer cells resulted in significant down-regulation of downstream ISGs (Fig. S5G). When sg*Nsun2* MC38 cancer cells were co-cultured with *Sucnr1*^−/−^ BMDMs derived from *Sucnr1* knockout mice, the expression of these downstream ISGs showed no significant differences compared with the experiment in which the cells were co-cultured with sg*Ctrl* MC38 cells, irrespective of *Nsun2*^MUT^ reintroduction in the rescue groups (Fig. 5H), indicating that NSUN2/GATA3-driven metabolic reprogramming of TCA in cancer cells modulates cGAS–STING signaling in macrophages through a noncanonical mechanism. To investigate the effect of NSUN2 on the TAM phenotype in vitro, TAM-like macrophages were generated by treating BMDMs with TCM derived from sg*Ctrl*, sg*Nsun2*, and r*Nsun2*^MUT^ cancer cells (Fig. 5I). Co-culturing splenic CD8^+^ T cells with TAMs treated with TCM from sg*Nsun2* MC38 cells significantly enhanced CD8^+^ T-cell cytotoxicity, evidenced by increased expression of IFN-γ and Gzmb, compared with that in the sg*Ctrl* group, whereas this effect was rescued by *Nsun2*^MUT^ (Fig. 5J and K and Fig. S5H). In contrast, co-culturing splenic CD8^+^ T cells with *Sucnr1*^−/−^TAMs treated with TCM from sg*Nsun2* MC38 cells did not result in any significant difference in CD8^+^ T-cell cytotoxicity (IFN-γ and Gzmb) compared with that in the WT group, as well as in the *Nsun2*^MUT^ rescue group (Fig. 5K and L and Fig. S5H). In summary, these findings indicate that succinate serves as an important bridge linking NSUN2-mediated immune evasion in cancer cells to the inhibition of the cGAS–STING signaling pathway in TAMs.

### Targeted degradation of NSUN2 enhanced response to ICB therapy

**Fig. 6.**
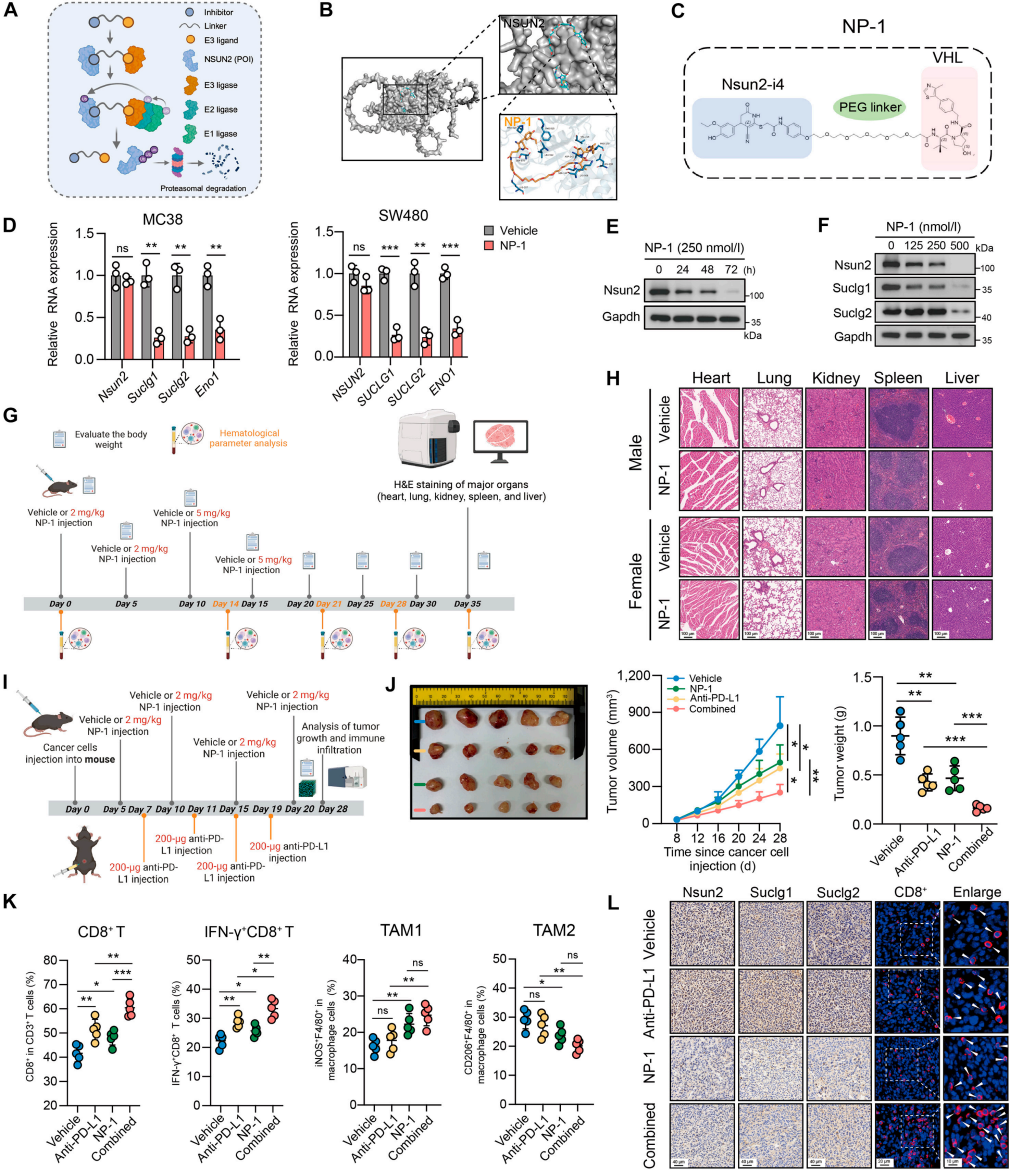
Targeted degradation of NSUN2 enhances the response to immune checkpoint blockade therapy. (A) Schematic illustration of the design strategy of NSUN2 PROTAC. (B) Predicted binding model of compound NP-1 with NSUN2. (C) Chemical structure of the PROTAC designated NP-1. (D) RT-qPCR analysis of *NSUN2*, *SUCLG1*, *SUCLG2*, and *ENO1* expression in vehicle- or NP-1-treated MC38 and SW480 cells. (E) WB analysis of the protein level of Nsun2 in MC38 cells treated with NP-1 at gradually escalating times. (F) WB analysis of the protein levels of Nsun2, Suclg1, and Suclg2 in MC38 cells treated with NP-1 at gradually escalating concentrations. (G) Schematic of NP-1 toxicity testing procedures. Male and female C57BL/6 mice aged 6 to 8 weeks were treated with NP-1 over 2 weeks under standard SPF conditions, following a sequential dose regimen: 2 mg/kg for the first 10 d and 5 mg/kg for the second 10 d. (H) Tissue histology of organs from NP-1-treated male and female mice at the experimental endpoint (day 35). (I) Schematic diagram of the animal model-based experiment for analyzing the immunotherapeutic effects of NP-1 and PD-L1. (J) Representative tumor photographs (left), tumor growth curves (middle), and final tumor weights (right) in C57BL/6 mice implanted with MC38 cells and treated with NP-1, anti-PD-L1, or the combination therapy (*n* = 5 per group). (K) Percentages of tumor-infiltrating CD8^+^ T cells, IFN-γ^+^CD8^+^ T cells, TAM1, and TAM2 macrophages in MC38-implanted tumors following the indicated treatments (*n* = 5 mice per group). (L) Representative IHC images of Nsun2, Suclg1, and Suclg2 expression and representative IF images of CD8^+^ T-cell infiltration in MC38-implanted tumors following the indicated treatments. Results are presented as mean ± SD. *P < 0.05; **P < 0.01; ***P < 0.001; ns, not significant through an unpaired *t* test. Abbreviations: ENO1, enolase 1; H&E, hematoxylin and eosin staining; IF, immunofluorescence; IFN-γ, interferon gamma; IHC, immunohistochemistry; MUT, mutant; NP-1, NSUN2-targeting PROTAC-1; NSUN2, NOP2/Sun RNA methyltransferase 2; PD-L1, programmed cell death ligand 1; PROTAC, proteolysis-targeting chimera; RT-qPCR, reverse transcription quantitative polymerase chain reaction; SD, standard deviation; SPF, specific-pathogen-free; SUCLG1, succinate-CoA ligase GDP/ADP-forming subunit α; SUCLG2, succinate-CoA ligase GDP/ADP-forming subunit β; TAM, tumor-associated macrophage; WB, western blot.

Based on our previous research [15] and the present study, NSUN2 promotes cancer progression via its classical function within cancer cells and facilitates immune evasion through nonclassical metabolic reprogramming. However, conventional small-molecule inhibitors only block the catalytic enzymatic activity of NSUN2 but fail to interfere with its noncanonical functions; accordingly, we designed PROTACs targeting NSUN2 to inhibit cancer progression and immune evasion by disrupting its dual function. As a novel modality in targeted therapy, PROTACs achieve specific elimination of target proteins by recruiting an E3 ubiquitin ligase to the protein of interest, thereby promoting ubiquitination and subsequent proteasomal degradation (Fig. 6A). In a previous study, we screened and characterized Nsun2-i4 as a specific NSUN2 ligand with verified inhibitory activity toward m^5^C methylation; this compound occupies the NSUN2 catalytic pocket, and its binding affinity has been confirmed via multiple biophysical assays [15]. Therefore, we hypothesized that Nsun2-i4 might be a suitable warhead for PROTAC construction. Molecular docking between NP-1 and the NSUN2 ligand-binding domain revealed that the carboxylic acid group was solvent-exposed, resulting in an ideal site for linker attachment (Fig. 6B). As a proof of concept, the NSUN2-targeting PROTAC NP-1 was synthesized by linking to a von Hippel–Lindau (VHL) E3 ligase ligand through a PEG linker via 2 amide bonds and subsequently evaluated in multiple cancer cell lines (Fig. 6C). NP-1 treatment did not affect *NSUN2* mRNA levels but significantly down-regulated those of its downstream targets, including *ENO1*, associated with its canonical function, and *SUCLG1/2*, linked to its noncanonical activity, across various cancer cell lines, such as MC38, SW480, and 4T1 cells (Fig. 6D and Fig. S6A). However, NP-1 administration markedly reduced the abundance of NSUN2 protein as the treatment duration and concentration increased, accompanied by a consistent down-regulation of the downstream proteins SUCLG1 and SUCLG2 in SW480 and MC38 cells (Fig. 6E and F and Fig. S6B and C). Among the 3 NSUN family members, NP-1 selectively reduced the NSUN2 protein levels without significantly affecting NSUN5 or NSUN6 (Fig. S6D). These findings demonstrate that NP-1 acts as an efficient NSUN2 degrader in vitro. The safety of NP-1 in both male and female C57BL/6J mice was evaluated in vivo by administering increasing doses up to 5 mg/kg (Fig. 6G). Complete blood counts, assessed at multiple time points, did not reveal significant alterations in hematological parameters, including total white blood cell, red blood cell, platelet, and lymphocyte counts (Fig. S6E). This observation may be explained by the fact that the NSUN2 expression was lower in whole blood than in other organs or tissues (Fig. S6F). After 35 d of treatment, no significant changes in the organ or body weights were observed (Fig. S6G and H). Moreover, H&E staining was used to assess the heart, lung, kidney, spleen, and liver tissues of male and female mice on day 35, which revealed that NP-1 treatment caused no damage to any of these organs (Fig. 6H). Mice were subcutaneously implanted with tumor cells on day 0, followed by intraperitoneal NP-1 (2 mg/kg) or vehicle treatment on days 5, 10, 15, and 20 and anti-PD-L1 antibody (200 μg/mouse) injection on days 7, 11, 15, and 19; tumor growth and immune infiltration were monitored periodically, and animals were euthanized for terminal analysis on day 28 (Fig. 6I). Both NP-1 and anti-PD-L1 treatment effectively inhibited tumor growth in the MC38 subcutaneous tumor model, whereas the combination therapy led to significantly greater tumor suppression than either of the treatments alone (Fig. 6J). Flow cytometry analysis revealed that NP-1 treatment increased the number of CD8^+^ T cells, cytotoxic CD8^+^ T cells, and TAM1 while reducing the infiltration of immunosuppressive TAM2, thereby reshaping the TME toward a more immunostimulatory phenotype (Fig. 6K). IHC and IF analyses revealed that NP-1 and combination therapy significantly reduced the NSUN2, SUCLG1, and SUCLG2 protein levels, whereas CD8^+^ T-cell infiltration markedly increased in all treatment groups (NP-1, anti-PD-L1, and most notably in the combination group), compared with that in the vehicle group (Fig. 6L and Fig. S6I and J). These findings offer valuable insights into the potential of the NSUN2-targeting PROTAC (NP-1) as an effective and safe immunotherapeutic strategy for clinical applications.

### Cancer patients with reduced NSUN2 expression exhibited favorable responses to cancer immunotherapies

**Fig. 7.**
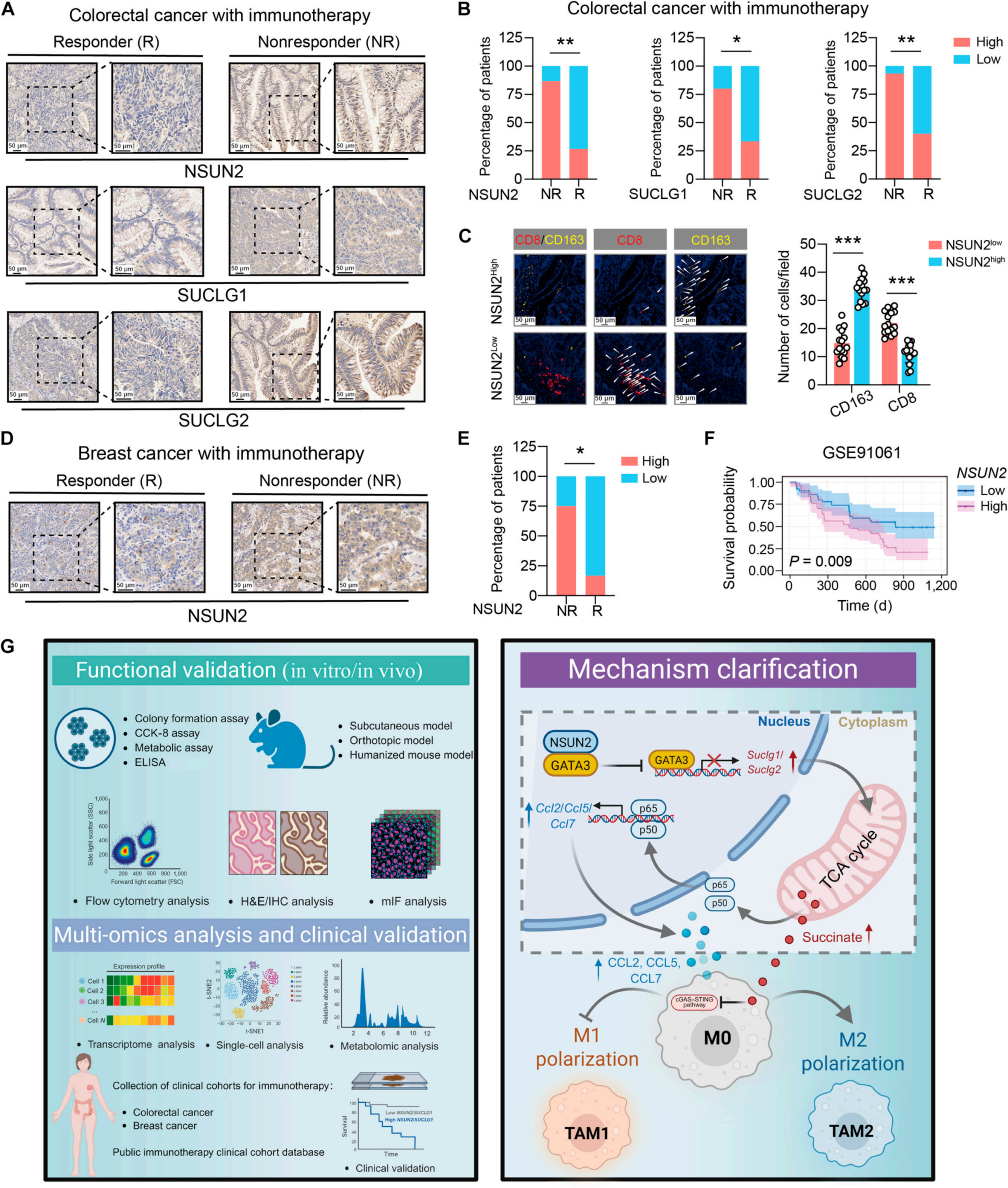
Cancer patients with low NSUN2 expression exhibit better responses to cancer immunotherapy. (A) Representative images from IHC staining of NSUN2 or SUCLG1 or SUCLG2 in responders (R) and nonresponders (NR) of 30 CRC patients with anti-PD-1 therapy from Zhongnan Hospital. (B) Statistical association between NSUN2 or SUCLG1 or SUCLG2 expression and response to anti-PD-1 therapy in 30 CRC patients. (C) Representative mIF images (left) of CD163^+^ and CD8^+^ cell staining, along with quantification (right) of CD163^+^ and CD8^+^ cells, in human CRC tissues with high (*n* = 15) or low (*n* = 15) NSUN2 expression. (D) Representative images of IHC staining of NSUN2 for responders (R) and nonresponders (NR) of human 30 BC patients with anti-PD-1 therapy. (E) Statistical association between NSUN2 expression and response to anti-PD-1 therapy in 30 BC patients. (F) Survival curve analysis of patients with NSUN2-high versus NSUN2-low expression in the immunotherapy dataset GSE91061 according to the TIGER database. (G) Schematic illustration of the noncanonical function of NSUN2 in modulating TAM polarization to facilitate cancer immune escape. Results are presented as mean ± SD. *P < 0.05; **P < 0.01; ***P < 0.001; ns, not significant through an unpaired *t* test. Abbreviations: BC, breast cancer; CRC, colorectal cancer; GSE, gene series expression dataset; IF, immunofluorescence; IHC, immunohistochemistry; mIF, multiplex immunofluorescence; NR, nonresponder; NSUN2, NOP2/Sun RNA methyltransferase 2; PD-1, programmed cell death 1; R, responder; SD, standard deviation; SUCLG1, succinate-CoA ligase GDP/ADP-forming subunit α; SUCLG2, succinate-CoA ligase GDP/ADP-forming subunit β; TAM, tumor-associated macrophage; TIGER, Tumor Immunotherapy Gene Expression Resource.

To explore the relationship between NSUN2 expression and immunotherapeutic efficacy, we performed IHC staining of specimens from patients with cancer who received immunotherapy. The results demonstrated that CRC patient samples from responders to anti-PD-1 therapy exhibited low NSUN2 expression accompanied by low SUCLG1 and SUCLG2 levels, whereas those from nonresponders showed high expression of NSUN2, SUCLG1, and SUCLG2 (Fig. 7A and B). To evaluate the clinical correlation between NSUN2 expression and immune cell infiltration in tumors, we first performed multiplex IF staining for CD163^+^ TAM2 macrophages and CD8^+^ T cells in CRC. CD8^+^ T cells significantly increased in NSUN2-low samples compared with those in NSUN2-high samples, whereas CD163^+^ TAM2 macrophages decreased (Fig. 7C). Similar results were observed in BC patients receiving immunotherapy, where IHC analyses showed that anti-PD-1 responders exhibited low NSUN2, SUCLG1, and SUCLG2 expression, whereas nonresponders displayed high levels of these proteins (Fig. 7D and E and Fig. S7A and B). Clinical cases from our center indicated that cancer patients with reduced NSUN2/SUCLG1/SUCLG2 expression exhibited favorable responses to cancer immunotherapies. Furthermore, analysis of the TIGER database indicated that high expression of NSUN2, SUCLG1, and SUCLG2 was associated with poorer prognosis compared to low expression, whereas elevated GATA3 levels correlated with improved outcomes across multiple immunotherapy cohorts (Fig. 7F and Fig. S7C). Similarly, interrogation of the Kaplan-Meier Plotter Immunotherapy database demonstrated that patients exhibiting high NSUN2 and SUCLG1 expression under PD-1/PD-L1/CTLA-4-based immunotherapy displayed significantly worse survival than those with low expression (Fig. S7D). From a clinical perspective, findings from our institutional data and public databases suggest that NSUN2 regulates the GATA3/SUCLG1/SUCLG2 axis through its moonlighting function, thereby reshaping the TME and correlating with immunotherapy sensitivity across diverse human cancers (Fig. 7G).

## Discussion

Immune evasion is a defining feature of cancer progression, enabling tumor cells to persist in the face of immune surveillance and contributing to treatment resistance and poor clinical outcomes. This growing challenge highlights the need to uncover additional therapeutic vulnerabilities that could be leveraged to improve immunotherapy efficacy. Increasing evidence has expanded the view of immune regulation in cancer beyond canonical signaling pathways, implicating posttranslational modifications of immune checkpoints, along with epigenetic rewiring and metabolic reprogramming, as key determinants of antitumor immune responses [11,28–30]. Within this framework, NSUN2, a widely expressed RNA methyltransferase, has been primarily studied for its established roles in tumorigenesis; however, whether NSUN2 also participates in broader, noncanonical regulatory circuits that shape tumor progression and immune escape remains an open and intriguing question. Through a series of assays for in vivo or in vitro confirmation, we identified NSUN2 as a key driver of cancer immune evasion independent of its catalytic activity (Fig. 1). Although gene amplification constitutes the predominant alteration of NSUN2 across multiple cancer types, mutations are nonetheless detected in a subset of tumors, including variants affecting the MTD. Beyond its oncogenic roles, NSUN2 acts as a host factor exploited by the Nipah virus to promote viral replication. The viral matrix protein stabilizes NSUN2 by preventing proteasomal degradation, while NSUN2 enhances matrix transcript stability through m^5^C modification of Nipah virus RNAs. Additionally, the noncatalytic domain of NSUN2 serves as a scaffold that recruits GNB2 and the E3 ligase TRIM28 to the matrix, promoting its ubiquitination and nuclear export to facilitate virion assembly [31]. However, the precise molecular mechanisms by which NSUN2 drives cancer immune evasion through its noncanonical function warrant further investigation.

Metabolic reprogramming is an important feature of cancer biology and immunology. Specific metabolites resulting from metabolic disorders in cancer cells not only drive tumor growth and metastasis but also impair the antitumor function of immune cells by modulating their function [32]. Transcriptomic and metabolomic analyses revealed that NSUN2 mediates metabolic reprogramming of the TCA cycle in cancer cells independent of its catalytic activity (Fig. 2). In particular, the NSUN2-driven accumulation of cancer-cell-derived succinate suppressed the cGAS–STING pathway in macrophages, promoting TAM2 polarization and enabling tumor immune evasion (Figs. 4 and 5). Thus, unraveling the determinants of the metabolic-disorder-associated immune landscape and its role in fueling cancer malignancy will provide a framework for developing effective therapeutic strategies against these highly prevalent diseases. Mechanistically, NSUN2 interacts with the transcription factor GATA3 to relieve its inhibitory effect on *SUCLG1* and *SUCLG2* expression, leading to increased succinate production (Fig. 3). GATA3, a member of the GATA family of transcription factors, plays a pivotal role in diverse physiological and pathological processes. By binding to the consensus DNA motif “GATA”, GATA3 regulates gene transcription across multiple tissues, including the immune, endocrine, and nervous systems [23]. In most contexts, GATA3 functions as a master transcriptional regulator that activates gene expression through direct DNA binding, chromatin remodeling, and coactivator recruitment [33,34], playing essential roles in Th2 cell differentiation [35,36], immune homeostasis[37], and tissue-specific development while frequently cooperating with factors such as UTX or forming complexes with MYB to drive transcriptional activation [33,34]. However, GATA3 exhibits versatile transcriptional functions; within breast epithelial cells, it promotes ERα66 transcription while repressing ERα36 expression, thereby maintaining a regulatory balance essential for normal mammary gland development that, when disrupted, may contribute to estrogen-receptor-positive BC progression, and it can also act directly as a transcriptional repressor, mediating doxorubicin resistance by inhibiting CYB5R2-catalyzed iron reduction in BC cells [23,38]. Interestingly, our study uncovers another noncanonical function of GATA3: it regulates metabolic reprogramming by transcriptionally repressing *SUCLG1* and *SUCLG2* (Figs. 2 and 3).

Targeting immunometabolism and integrating it with immunotherapy is expected to offer a practical strategy for creating a “catfish effect” helping to overcome metabolic barriers and enhance immunotherapeutic efficacy. However, no clinically approved therapies targeting the noncanonical pathway of NSUN2 are currently available. Numerous studies, including ours, have demonstrated that NSUN2 regulates tumorigenesis and metastasis in various cancers through its classical enzymatic activity, by modulating NSUN2/ENO1 and multiple other pathways. Thus, in terms of application, the novel PROTAC developed in this study targets NSUN2 for degradation via the ubiquitin–proteasome pathway. This therapy not only eliminates NSUN2-mediated tumorigenesis through its classical m^5^C-dependent function but also reshapes the TME by inhibiting TAM2 polarization, independent of its catalytic activity (Fig. 6). However, under the same treatment regimen, NP-1 demonstrates an excellent safety profile in vivo, likely because of its high specificity as a PROTAC targeting NSUN2, which minimizes off-target toxicity. Moving forward, we will advance preclinical investigations to unlock its full translational potential for future clinical development.

Meanwhile, the present study had several limitations. First, a potential limitation of NP-1 is its reliance on VHL as an E3 ligase, given that VHL is a well-known tumor suppressor that is frequently mutated in human cancers, such as renal cell carcinomas [39]. Second, a further important consideration is that NSUN2 exerts essential physiological functions beyond tumor biology, including the regulation of fetal brain development, as evidenced by developmental abnormalities in *Nsun2*-deficient models, and the promotion of genome stability through facilitating DICER-mediated cleavage of DNA-damage-associated R-loops [40,41]. Accordingly, direct therapeutic targeting of NSUN2 with small-molecule inhibitors or PROTAC-based strategies may entail a risk of on-target toxicity. Future studies will therefore prioritize the development of tumor-selective targeting approaches to mitigate potential adverse effects and facilitate clinical translation.

## Conclusions

Our findings indicate that NSUN2 promotes cancer immune evasion through its moonlighting role in TCA cycle metabolic reprogramming. Of note, this study serves as an extension of our previous research, highlighting the important role of NSUN2 in reshaping the TME, independent of its m^5^C RNA modification activity. Moreover, we developed an effective PROTAC that targets NSUN2 and eliminates its dual functions; targeted degradation of NSUN2 activated the cGAS–STING innate immune pathway in macrophages, thereby inhibiting TAM2 polarization and enhancing antitumor immunity. Our findings suggest that targeting NSUN2 may enhance the response of cancer patients to PD-L1 blockade, offering a promising therapeutic strategy for clinical oncology.

## Ethical Approval

This study was approved by Zhongnan Hospital’s Ethics Committee (approval number: 2025094K). The tissue samples were obtained with written informed consent from each patient. All animal procedures were approved by the Animal Care Committee of the Wuhan Institute of Virology, Chinese Academy of Sciences (approval number: WIVA04202301).

## Data Availability

The data and materials are available from the corresponding authors upon reasonable request. Bulk RNA-seq data for this study have been deposited in the National Genomics Data Center, China National Center for Bioinformation/Beijing Institute of Genomics, Chinese Academy of Sciences (https://ngdc.cncb.ac.cn/bioproject/), with accession numbers PRJCA057716 and PRJCA057660.
