# Supplementary material for: NSUN2 Promotes Cancer Immune Evasion via Its Moonlighting Function Acting on Metabolic Reprogramming
Source: Cancer Commun (Lond). 2026 Aug 19;46:0042. doi: 10.34133/cancomm.0042 (PMC13486727; doi:10.34133/cancomm.0042)
Supplement: Supplementary file 1 [file cancomm.0042.f1.pdf]

NSUN2 Promotes Cancer Immune Evasion via its Moonlighting Function Acting on Metabolic Reprogramming

Baoxiang Chen[1,2,3,4,5,†,*], Yanrong Deng[1,2,3,4,†], Xiang Zhai[1,2,3,4,†], Peiyun Wang[1,2,3,4,6,†], Kunming Lei[1,2,3,4], Siyuan Yin[1,2,3,4], Quanjiao Chen[7], Qun Qian[1,2,3,4], Jinfang Zhang[8,9], Xianghai Ren[1,2,3,4,*], Jianhong Zhao[1,2,3,4,*], Congqing Jiang[1,2,3,4,*]

Affiliations:

[1]Hubei Key Laboratory of Intestinal and Colorectal Diseases, Zhongnan Hospital of Wuhan University, Wuhan, China.

[2]Clinical Center of Intestinal and Colorectal Diseases of Hubei Province, Zhongnan Hospital of Wuhan University, Wuhan, China.

[3]Department of Colorectal and Anal Surgery, Zhongnan Hospital of Wuhan University, Wuhan, China.

[4]Low Rectal Cancer Diagnosis and Treatment Center, Zhongnan Hospital of Wuhan University, Wuhan, China.

[5]Rosalind and Morris Goodman Cancer Institute, McGill University, Montreal, QC , Canada.

[6]Department of Colorectal and Anal Surgery, Jingzhou Hospital Affiliated to Yangtze University, Jingzhou, China.

[7]CAS Key Laboratory of Special Pathogens and Biosafety, Chinese Academy of Sciences, Wuhan, China.

[8]Frontier Science Center of Immunology and Metabolism, Medical Research Institute, Wuhan University, Wuhan, Hubei, P. R. China.

[9]State Key Laboratory of Metabolism and Regulation in Complex Organisms, College of Life Sciences, Taikang Center for Life and Medical Sciences, Wuhan University, Wuhan, China.

[†]Baoxiang Chen, Yanrong Deng, Xiang Zhai, and Peiyun Wang contributed equally to this work and share first authorship contributed equally to this work.

[#]Corresponding authors: Congqing Jiang (wb002554@whu.edu.cn), Baoxiang Chen (bxchenmcgill@gmail.com), Jianhong Zhao (19jhzhao@whu.edu.cn) and Xianghai Ren (xhren@whu.edu.cn).

Supplementary Figures

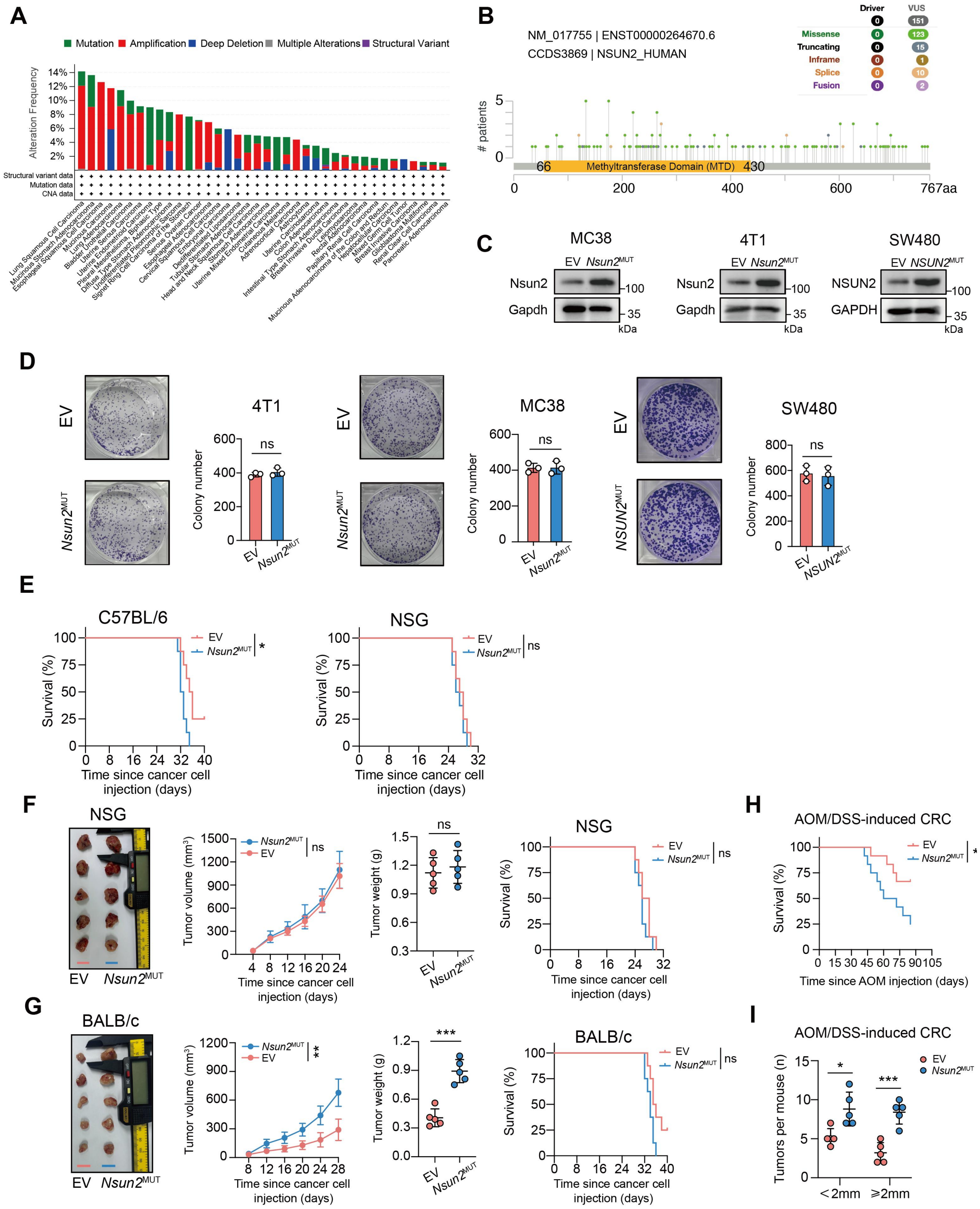

Supplementary Figure S1. NSUN2 functions as a driver of cancer immune evasion independent of the catalytic activity.

(A) Summary of genomic alterations of NSUN2 across diverse cancer types using the cBioPortal database. Deep deletion (blue) indicates homozygous loss of the gene, typically resulting in loss of gene function. **(B)** Summary of NSUN2 somatic mutation

frequency across the full-length protein based on data from the cBioPortal database, covering 151 somatic mutations from 132 patients.**(C)** WB analysis of indicated protein levels in EV and *NSUN2*^{MUT} 4T1, MC38, SW480 cancer cells. **(D)** Colony formation assay for EV and *NSUN2*^{MUT} 4T1, MC38, SW480 cells. $n = 3$ biologically independent samples. **(E)** Kaplan-Meier survival curves of immunocompetent C57BL/6J mice or immunodeficient NSG mice bearing EV and *Nsun2*^{MUT} MC38 cells. **(F)** Representative images of excised tumors, tumor growth curves, endpoint tumor weight, and Kaplan-Meier survival analysis of immunodeficient NSG mice bearing EV and *Nsun2*^{MUT} 4T1 cells. **(G)** Representative images of excised tumors, tumor growth curves, endpoint tumor weight, and Kaplan-Meier survival analysis of immunocompetent BLAB/c mice bearing EV and *Nsun2*^{MUT} 4T1 cells. **(H)** The survival curve of EV and *Nsun2*^{MUT} mice in AOM/DSS-induced orthotopic CRC model. **(I)** Comparison of the indicated size of CRC numbers in mice from EV and *Nsun2*^{MUT} groups. Results are presented as the mean ± SD. $^{*}P < 0.05$, $^{**}P < 0.01$, $^{***}P < 0.001$; ns, no significant. Abbreviations: AOM, Azoxymethane; CRC, Colorectal cancer; DSS, Dextran sulfate sodium; EV, Empty vector; NSG, NOD-*Prkdc*^{scid}-*Il2rg*^{null} mice; NSUN2/Nsun2, NOP2/Sun RNA methyltransferase 2; SD, Standard deviation; VUS, Variant of uncertain significance; WB, Western blot.

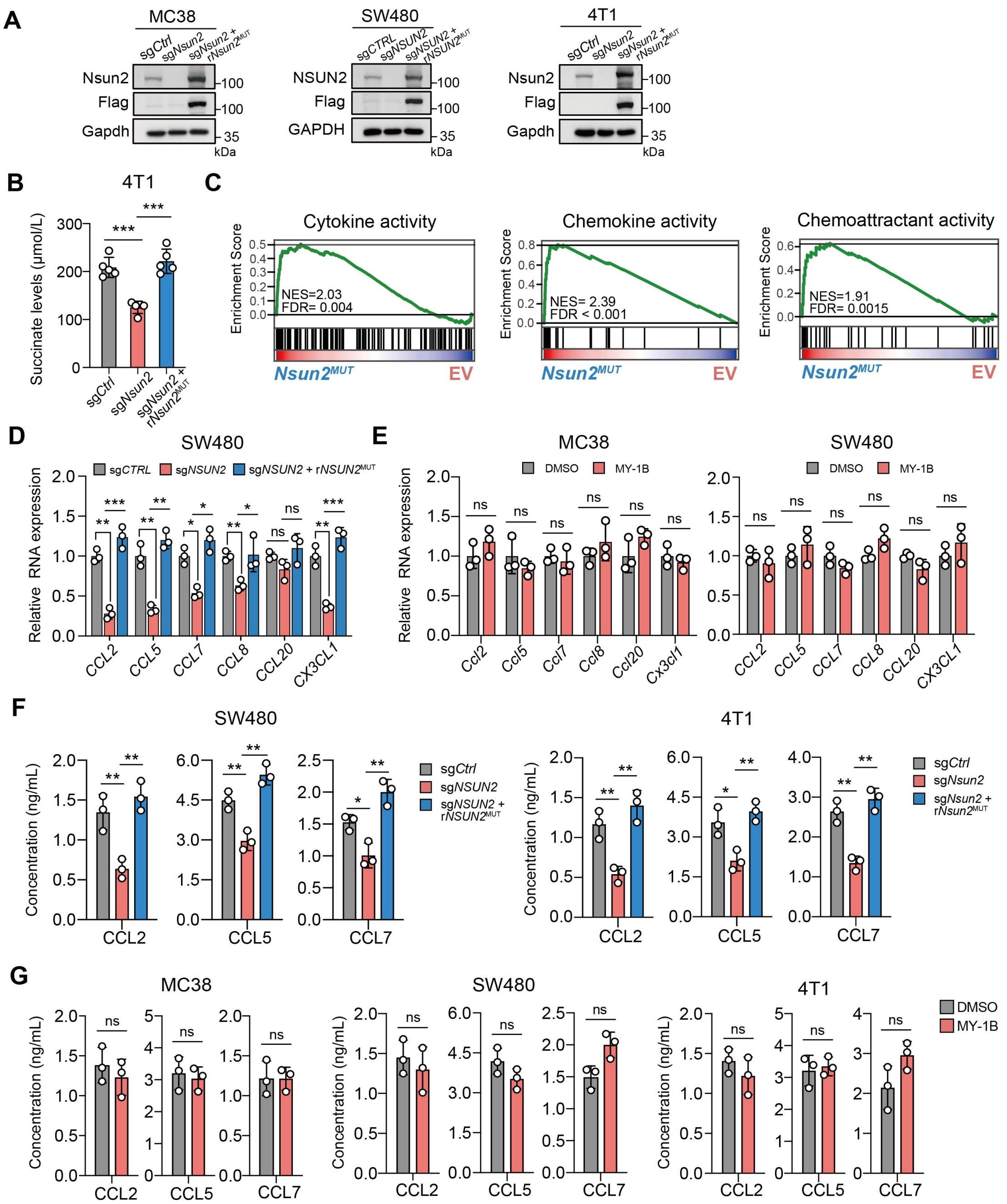

Supplementary Figure S2. NSUN2 promotes TCA metabolic reprogramming and chemokine production independently of the catalytic activity

(A) WB analysis of the indicated protein levels in sg*Ctrl*, sg*Nsun2*, sg*Nsun2* + r*Nsun2*^{MUT} MC38, SW480, and 4T1 cancer cell lines. **(B)** Determination of succinate levels in sg*Ctrl*, sg*Nsun2*, and *Nsun2*^{MUT}-reconstituted 4T1 cells. **(C)** GSEA analysis to assess specific enrichment of cytokine, chemoattractant, and chemokine activities in tumor tissues from MC38 *Nsun2*^{MUT} and EV groups based on KEGG pathway dataset. **(D)** RT-qPCR analysis of the expression of several chemokine-associated

genes in sg*Ctrl*, sg*NSUN2*, and *NSUN2*^{MUT} -reconstituted SW480 cells. **(E)** RT-qPCR analysis of the expression of several chemokine-associated genes in MC38 or SW480 cells treated with DMSO or 5 μmol/L MY-1B. **(F)** ELISA analysis of the CCL2, CCL5, and CCL7 cytokine levels from sg*Ctrl*, or, sg*Nsun2,* or r*Nsun2*^{MUT} SW480 (left) and 4T1 cells (right). **(G)** ELISA analysis of the CCL2, CCL5, and CCL7 cytokine levels from MC38 (left), SW480 (middle) and 4T1 (right) cells treated with DMSO or 5 μmol/L MY-1B. Results are presented as the mean ± SD. *$P < 0.05$, **$P < 0.01$, ***$P < 0.001$; ns, no significant. Abbreviations: CCL2/5/7, C-C motif chemokine ligand 2/5/7; DMSO, Dimethyl sulfoxide; ELISA, Enzyme-linked immunosorbent assay; EV, Empty vector; GSEA, Gene Set Enrichment Analysis; KEGG, Kyoto Encyclopedia of Genes and Genomes; NSUN2/Nsun2, NOP2/Sun RNA methyltransferase 2; RT-qPCR, Reverse transcription quantitative real-time polymerase chain reaction; SD, Standard deviation; sgCtrl, Single-guide RNA control; TCA, Tricarboxylic acid cycle; WB, Western blot.

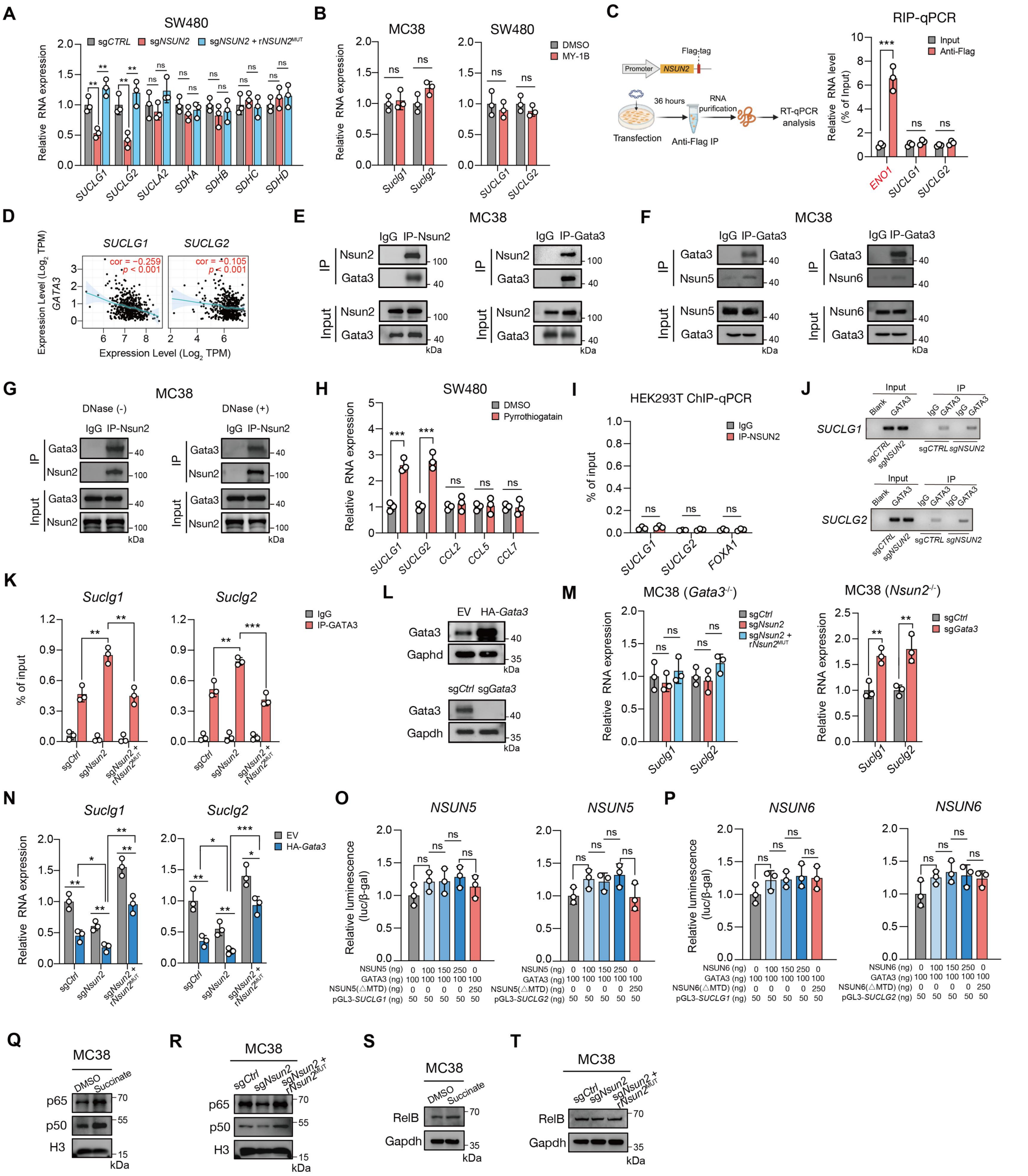

Supplementary Figure S3. NSUN2 promoted TCA metabolic reprogramming and chemokines production independent of the catalytic activity

(A) RT-qPCR analysis of the succinate-related metabolic genes expression level from sg*CTRL*, sg*NSUN2*, and *NSUN2*^{MUT} SW480 cells, n = 3 samples for each group. **(B)** RT-qPCR analysis of the succinate-related genes expression in MC38 or SW480 cells treated with DMSO or 5 µmol/L MY-1B, n = 3 samples for each group. **(C)** Schematic representation of the RIP-qPCR assay and RIP-qPCR analysis of the indicated genes in the anti-Flag immunoprecipitates. **(D)** Correlation analysis of the expression of *GATA3* and *SUCLG1* or *SUCLG2* in TCGA-COAD and TCGA-READ

database. **(E)** Endogenous IP analysis of the interaction between Gata3 and Nsun2 from MC38 cells. **(F)** Endogenous IP analysis of the interaction between Gata3 and Nsun5 or Nsun6 from MC38 cells. **(G)** Endogenous IP analysis of the interaction between GATA3 and NSUN2 in MC38 cells with or without DNase I treatment. **(H)** RT-qPCR analysis of the indicated genes in SW480 treated with DMSO or pyrrothiogatain. **(I)** ChIP-qPCR analysis of NSUN2 enrichment at the promoters of GATA3 downstream target genes *SUCLG1*, *SUCLG2*, and *FOXA1* in HEK293T cells. Three primer pairs were designed to cover the distal (-1500 to -1000), mid-promoter (-1000 to -500), and core promoter (-300 to +100) regions of these genes. **(J)** ChIP analysis by electrophoresis in sg*CTRL* and sg*NSUN2* SW480 cells. **(K)** ChIP-qPCR analysis of the enrichment of *Suclg1* and *Suclg2* in sg*Ctrl*, sg*Nsun2*, and sg*Nsun2*+ r*Nsun2*^{MUT} MC38 cells. **(L)** WB validation of *Gata3* knockout or overexpression in MC38 cells. **(M)** RT-qPCR analysis of *Suclg1* and *Suclg2* mRNA expression in *Gata3* knockout MC38 cells with sg*Ctrl*, sg*Nsun2*, and sg*Nsun2*+ r*Nsun2*^{MUT}, as well as in *Nsun2* knockout MC38 cells with carrying EV or *Gata3* overexpression plasmid.**(N)** RT-qPCR analysis of *Suclg1* and *Suclg2* mRNA levels in sg*CTRL*, sg*NSUN2*, and *NSUN2*^{MUT}-reconstituted MC38 cells following *Gata3* overexpression or EV control. **(O)** Luciferase reporter analysis of the transcriptional activation of *SUCLG1* and *SUCLG2* mediated by *NSUN5* or its ΔMTD variants. **(P)** Luciferase reporter analysis of the transcriptional activation of *SUCLG1* and *SUCLG2* mediated by *NSUN6* or its ΔMTD variants. **(Q)** Western blot analysis of nuclear p50 and p65 protein levels in cells treated with succinate treatment. **(R)** Western blot analysis of nuclear p50 and p65 protein levels in sg*Ctrl*, sg*Nsun2* or r*Nsun2*^{MUT} cells. **(S)** Western blot analysis of RelB protein level in cells treated with succinate or DMSO. **(T)** Western blot analysis of Relb protein levels in sg*Ctrl*, sg*Nsun2* or sg*Nsun2* + r*Nsun2*^{MUT} cells. Results are presented as the mean ± SD. $*P < 0.05$, $**P < 0.01$, $***P < 0.001$; ns, no significant. Abbreviations: ChIP-qPCR, Chromatin immunoprecipitation quantitative real-time polymerase chain reaction; DNase I, Deoxyribonuclease I; DMSO, Dimethyl sulfoxide; EV, Empty vector; FOXA1, Forkhead box A1; GATA3/Gata3, GATA binding protein 3; IP, Immunoprecipitation;

MTD, Methyltransferase domain; NSUN2/Nsun2, NOP2/Sun RNA methyltransferase 2; NSUN5/Nsun5, NOP2/Sun RNA methyltransferase 5; NSUN6/Nsun6, NOP2/Sun RNA methyltransferase 6; p50, NF-κB p50 subunit; p65, NF-κB p65 subunit; RIP-qPCR, RNA immunoprecipitation quantitative real-time polymerase chain reaction; RT-qPCR, Reverse transcription quantitative real-time polymerase chain reaction; SD, Standard deviation; sgCtrl/sgCTRL, Single-guide RNA control; SUCLG1/Suclg1, Succinyl-CoA ligase gamma subunit α; SUCLG2/Suclg2, Succinyl-CoA ligase gamma subunit beta; TCGA, The Cancer Genome Atlas; TCGA-COAD, TCGA colon adenocarcinoma dataset; TCGA-READ, TCGA rectum adenocarcinoma dataset; WB, Western blot.

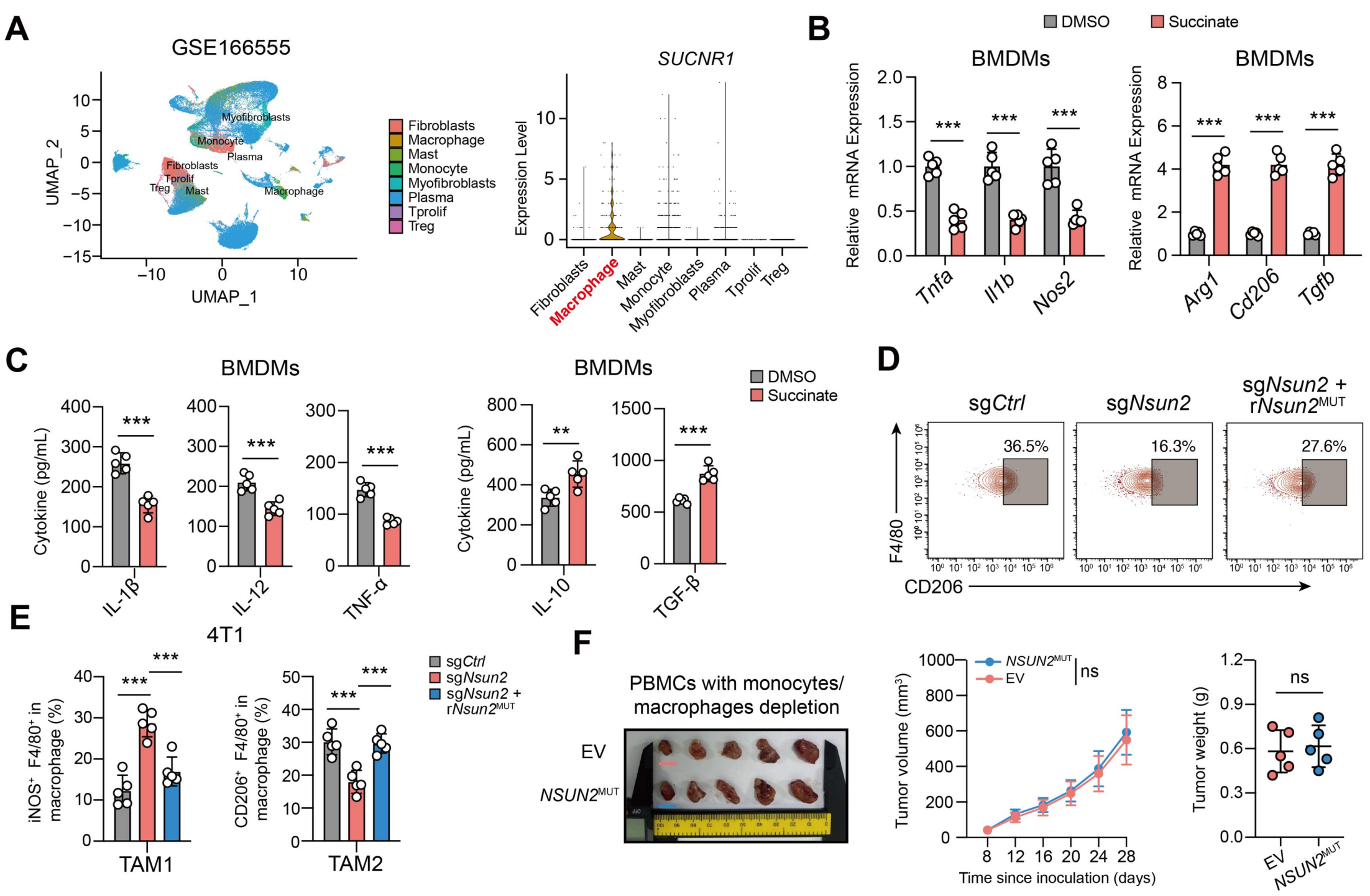

Supplementary Figure S4. NSUN2-mediated elevation of succinate orchestrated TAM reprogramming and facilitated immune evasion

(A) Analysis of the *SUCNR1* expression across different immune cell types in 12 CRC patients using the GSE166555 database. **(B)** RT-qPCR analysis of the expression levels of TAM1-associated or TAM2-associated genes in BMDMs treated with succinate compared to those treated with DMSO. **(C)** ELISA analysis of the

IL-1β, IL-12, and TNF-α, IL-10 and TGF-β cytokine level from BMDMs cells treated with DMSO or succinate. **(D)** Representative plots and percentages of iNOS$^+$F4/80$^+$ cells from co-culture sg*Ctrl*, sg*Nsun2*, and r*Nsun2*^{MUT} MC38 cells with RAW264.7 cells. **(E)** Statistical analysis of iNOS$^+$ F4/80$^+$ or CD206$^+$ F4/80$^+$ cells from co-culture sg*Ctrl*, sg*Nsun2*, and r*Nsun2*^{MUT} 4T1 cells with RAW264.7 cells using FACS, $n = 5$ per group. **(F)** Representative tumor images (left), tumor growth curves (middle), and endpoint tumor weight (right) of NOG mice bearing *NSUN2*^{MUT} or control SW480 tumors treated with PBMCs depleted of monocytes/macrophages ($n = 5$ per group). Results are presented as the mean ± SD. *$P < 0.05$, **$P < 0.01$, ***$P < 0.001$; ns, no significant. Abbreviations: BMDMs, Bone marrow-derived macrophages; CRC, Colorectal cancer; DMSO, Dimethyl sulfoxide; ELISA, Enzyme-linked immunosorbent assay; FACS, Fluorescence-activated cell sorting; GSE, Gene Expression Series; IL-1β, Interleukin-1 beta; IL-10, Interleukin-10; IL-12, Interleukin-12; iNOS, Inducible nitric oxide synthase; NOG, NOD.Cg-*Prkdc*^{scid}*Il2rg*^{tm1Sug}/ShiJic mice; NSUN2, NOP2/Sun RNA methyltransferase 2; PBMCs, Peripheral blood mononuclear cells; RT-qPCR, Reverse transcription quantitative real-time polymerase chain reaction; SD, Standard deviation; sgCtrl, Single-guide RNA control; SUCNR1, Succinate receptor 1; TAM, Tumor-associated macrophage; TGF-β, Transforming growth factor-beta; TNF-α, Tumor necrosis factor-alpha.

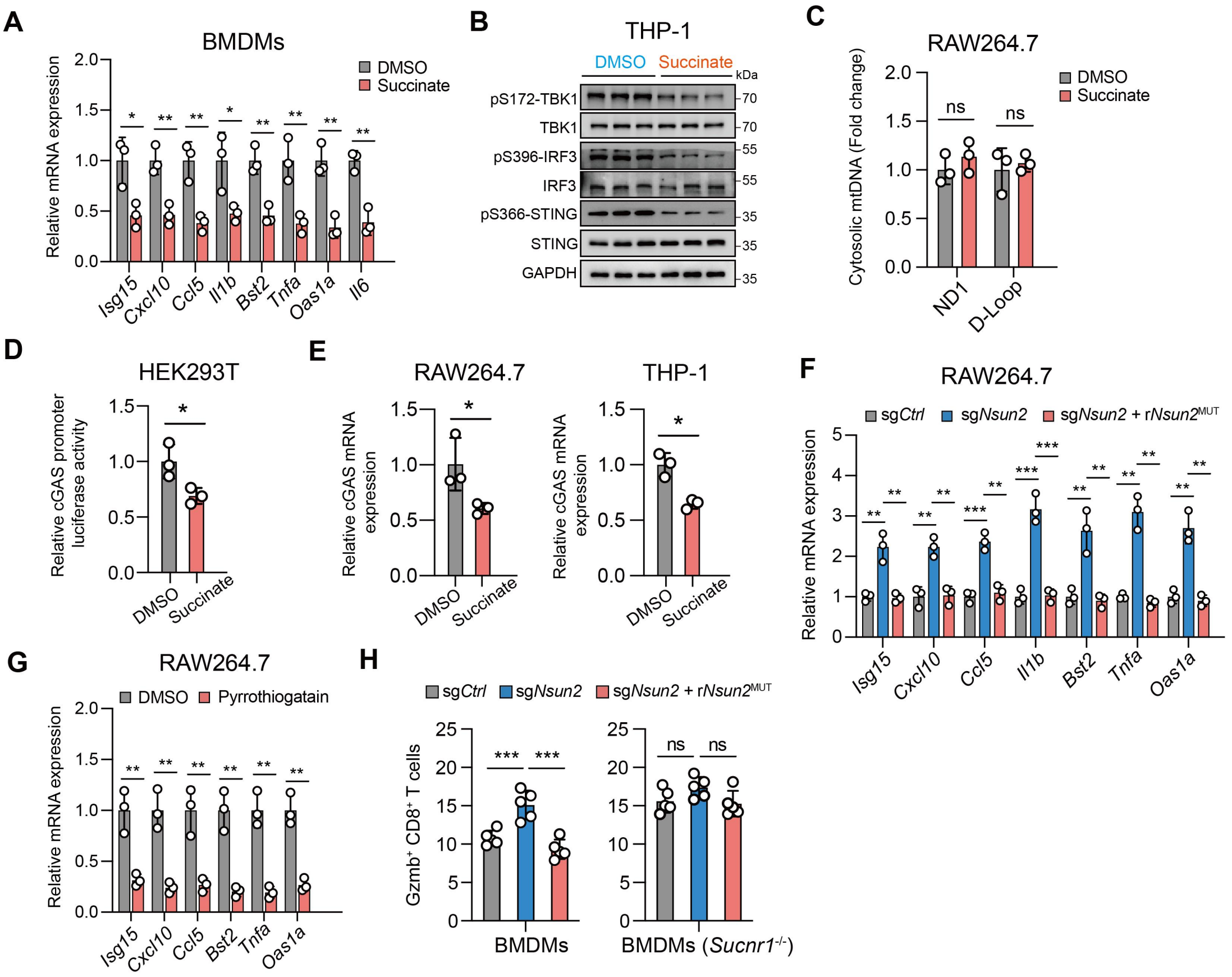

Supplementary Figure S5. Succinate orchestrates TAMs reprogramming and immune evasion via cGAS-STING pathway.

(A) RT-qPCR analysis of the cytosolic DNA-sensing pathway gene expression level from BMDMs cells treated with succinate compared to those treated with DMSO. **(B)** WB analysis of the indicated protein levels in THP-1 cells treated with succinate. **(C)** Quantification of mtDNA (*D-loop* and *ND1*) by RT-qPCR in RAW264.7 treated with succinate for 24 h. n = 3 samples for each group. **(D)** Luciferase activity on cGAS promoter in HEK293T cells treated with succinate or DMSO. **(E)** Relative mRNA of cGAS in RAW264.7 (left) and THP-1 (right) cells treated with succinate or DMSO. **(F)** RT-qPCR analysis of the expression levels of interferon-stimulated genes from RAW264.7 co-cultured with sg*Ctrl*, sg*Nsun2,* or sg*Nsun2* + r*Nsun2*^{MUT} MC38 cells. **(G)** RT-qPCR analysis of the expression levels of interferon-stimulated genes from RAW264.7 co-cultured MC38 cells treated with DMSO or pyrrothiogatain. **(H)** Statistical analysis tumor-infiltrating GzmB^{+} CD8^{+} T cells from FACS, derived from co-culture of sg*Ctrl*, sg*Nsun2*, and sg*Nsun2* + r*Nsun2*^{MUT} MC38 cells with BMDMs, n

= 5 per group. Results are presented as the mean ± SD. $*P < 0.05$, $**P < 0.01$, $***P < 0.001$; ns, no significant. Abbreviations: BMDMs, Bone marrow-derived macrophages; cGAS, Cyclic GMP-AMP synthase; DMSO, Dimethyl sulfoxide; GzmB, Granzyme B; mtDNA, Mitochondrial DNA; ND1, Mitochondrial NADH dehydrogenase subunit 1; Nsun2/NSUN2, NOP2/Sun RNA methyltransferase family member 2; RT-qPCR, Reverse transcription quantitative real-time polymerase chain reaction; SD, Standard deviatio

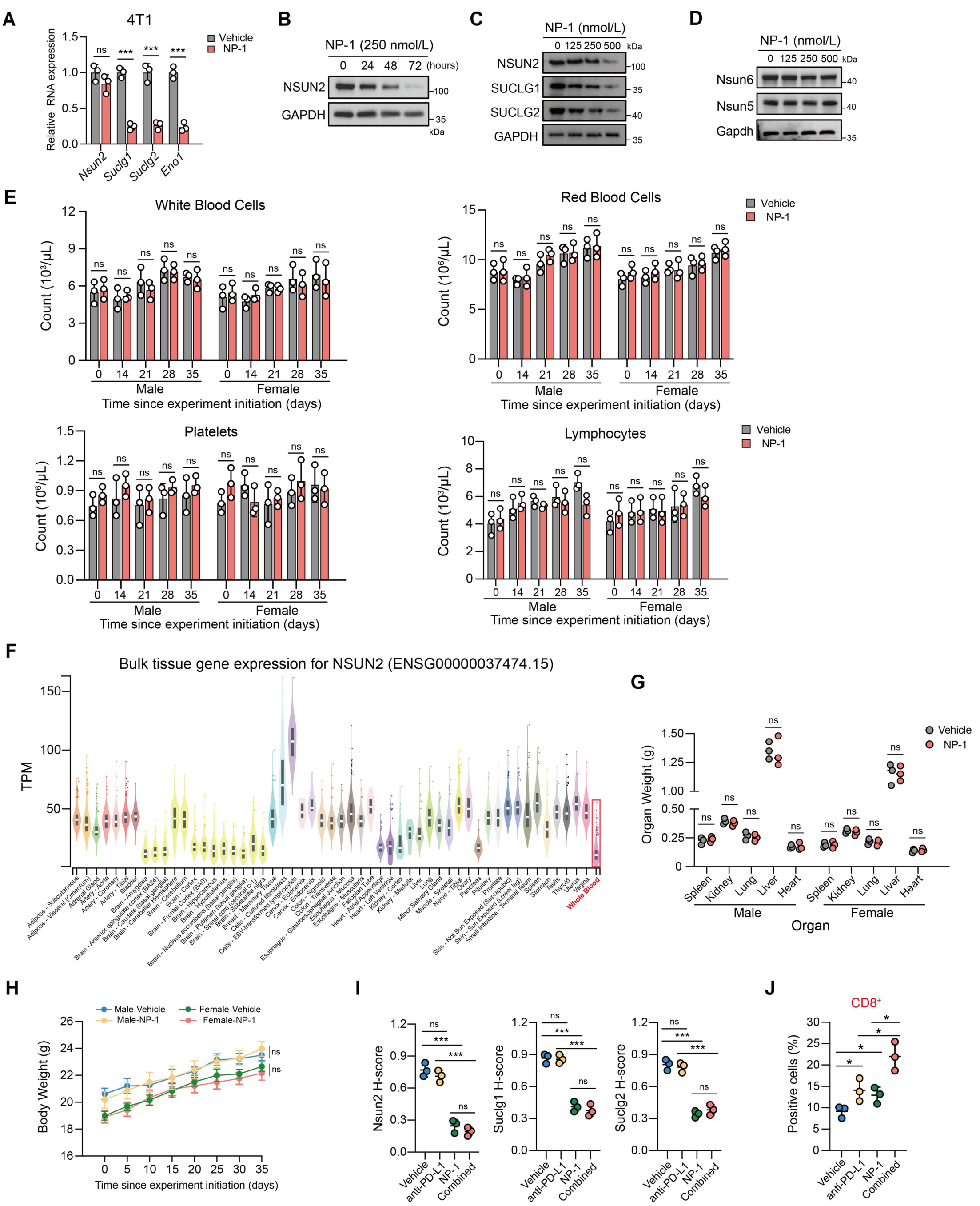

Supplementary Figure S6. Targeted degradation of NSUN2 enhances response to immune checkpoint blockade therapy.

(A) RT-qPCR analysis of the expression of *Nsun2*, *Suclg1*, *Suclg2*, and *Eno1* in vehicle or NP-1 treatment 4T1 cells. **(B)** WB analysis of the NSUN2 protein level in SW480 cells treated with NP-1 at gradually escalating times. **(C)** WB analysis of the protein levels of NSUN2, SUCLG1 and SUCLG2 in SW480 cells treated with NP-1 at gradually escalating concentrations. **(D)** WB analysis of the Nsun5 and Nsun6 proteins level in MC38 cells treated with NP-1 at gradually escalating concentrations.

(E) Hematology analysis of different blood cell components after NP-1 or vehicle treatment, including white blood cells, red blood cells, platelets, and lymphocytes. **(F)** The NSUN2 gene expression level in different human normal organs and tissues was displayed by utilizing GTEx portal website. Expression values are shown in TPM (Transcripts Per Million), calculated from a gene model with isoforms collapsed to a single gene. **(G)** Organ weight analysis of NP-1-treated male and female mice, evaluated after 35 days of treatment. **(H)** Body weight analysis of male and female mice treated with NP-1 over a 35-day period (measured every five days). **(I)** Protein expression levels of Nsun2, Suclg1, and Suclg2 were quantified using H-scores derived from DAB staining intensity (data from Figure 6L). **(J)** Statistical analysis of the percentage of $CD8^+$ T cells in the indicated groups (data from Figure 6L). Results are presented as the mean ± SD. **P* < 0.05, ***P* < 0.01, ****P* < 0.001; ns, no significant. Abbreviations: DAB, 3,3'-Diaminobenzidine; Eno1, Enolase 1; GTEx, Genotype-Tissue Expression; H-score, Histochemical score; Nsun2, NOP2/Sun RNA methyltransferase 2; Nsun5, NOP2/Sun RNA methyltransferase 5; Nsun6, NOP2/Sun RNA methyltransferase 6; NP-1, NSUN2 PROTAC-1; RT-qPCR, Reverse transcription quantitative real-time polymerase chain reaction; SD, Standard deviation; SUCLG1/Suclg1, Succinyl-CoA ligase gamma subunit α; SUCLG2/Suclg2, Succinyl-CoA ligase gamma subunit beta; TPM, Transcripts Per Million; WB, Western blot.

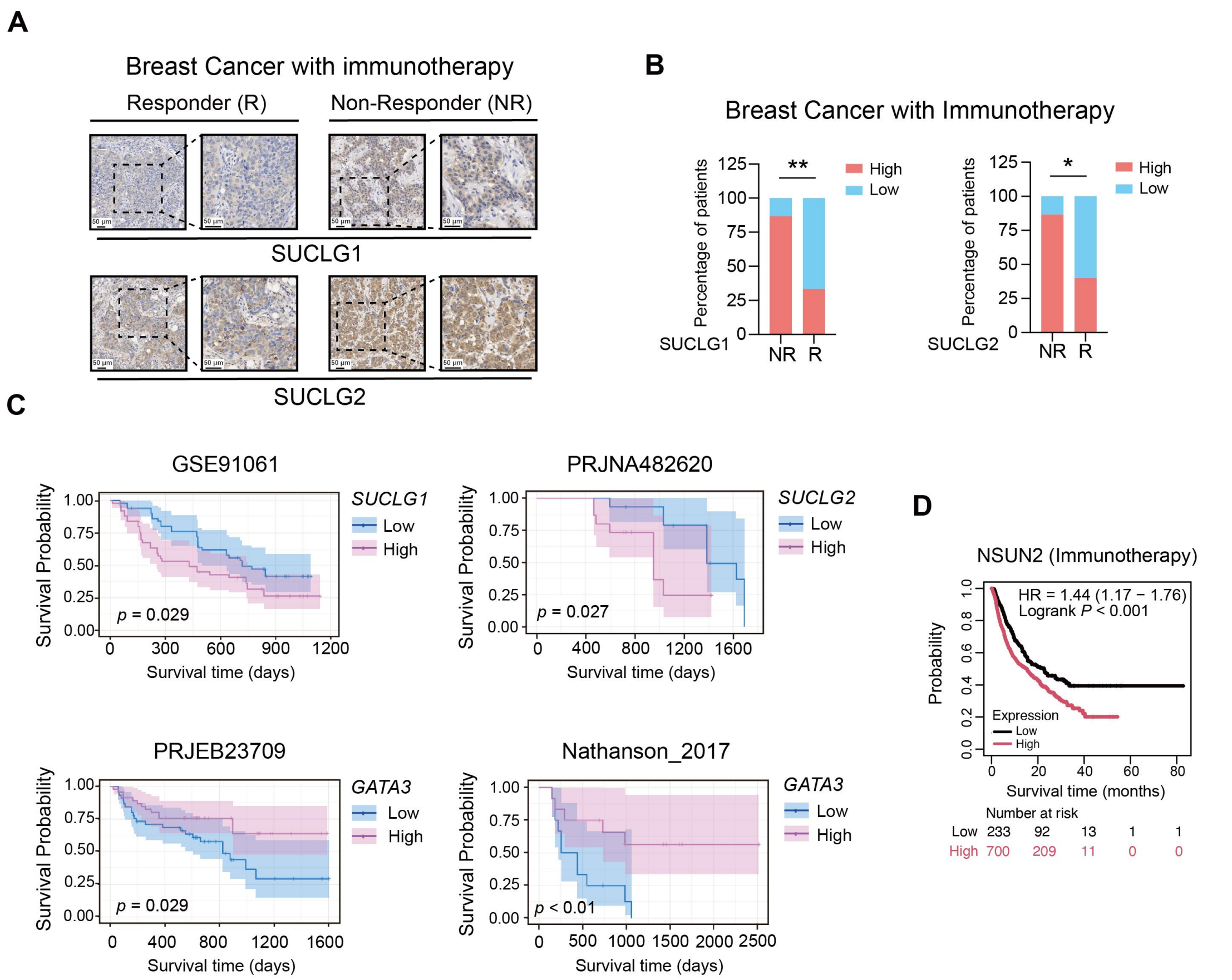

Supplementary Figure S7. Cancer patients with low NSUN2 expression exhibit better responses to cancer immunotherapy.

(A) Representative images from IHC staining of SUCLG1 or SUCLG2 in responders (R) and non-responders (NR) of 30 BC patients with anti-PD-1 therapy. **(B)** Statistical association between SUCLG1 or SUCLG2 expression and response to anti-PD-1 therapy in BC patients. **(C)** Survival analyses of immunotherapy-treated patients with high versus low SUCLG1 (GSE91061), SUCLG2 (PRJNA482620), and GATA3 expression (PRJEB23709 and Nathanson_2017) based on the TIGER database. **(D)** Kaplan–Meier survival analyses of immunotherapy-treated patients with high versus low NSUN2 expression using the Kaplan-Meier Plotter Immunotherapy database (n= 933). Abbreviations: anti-PD-1, Programmed death-1 antibody; BC, Breast cancer; GSE, Gene Expression Series; GATA3, GATA binding protein 3; IHC, Immunohistochemistry; NR, Non-responders; NSUN2, NOP2/Sun RNA; SUCLG1/ Suclg1, Succinyl-CoA ligase gamma subunit α; SUCLG2/Suclg2, Succinyl-CoA ligase gamma subunit beta.

Supplementary Tables

Supplementary Table S1. Primer sequences and sgRNAs used in this study.

**Primer sequences (5'→3')**	
**sgRNA sequences**	
sg*Ctrl*-mouse	CTTCACGCCTTGGACCGATA
sg*Nsun2*-mouse	GCTACAGGTCTACCGAGAC
sg*Gata3*-mouse	GCTACAGGTCTACCGAGAC
sg*CTRL*-human	AAAGAAAGAGGAATAGTAGC
sg*NSUN2*-human	GCTGTTCGAGCACTACTACC
**Primers for RT-qPCR (mouse genes)**	
Ccl2-PF	TTAAAAACCTGGATCGGAACCAA
Ccl2-PR	GCATTAGCTTCAGATTTACGGGT
Ccl5-PF	GCTGCTTTGCCTACCTCTCC
Ccl5-PR	TCGAGTGACAAACACGACTGC
Ccl7-PF	GCTGCTTTCAGCATCCAAGTG
Ccl7-PR	CCAGGGACACCGACTACTG
Ccl8-PF	TCTACGCAGTGCTTCTTTGCC
Ccl8-PR	AAGGGGGATCTTCAGCTTTAGTA
Ccl20-PF	ATGGCCGATGAAGCTTGTGA
Ccl20-PR	CTCCTTGGGCTGTGTCCAAT
Cx3cl1-PF	CTACTAGGAGCTGCGACACG
Cx3cl1-PR	CTTGGGAAGTCCCCATGGTC
Suclg1-PF	TCAGCAGGCTTTGGAGTACG
Suclg1-PR	TGAATGTGGCCAGGCATGAT
Suclg2-PF	AACACTCCAAACTCTCGGCA
Suclg2-PR	TTCACTTCCACCTGAGTGGC
Sucla2-PF	ACGGGTCGGACTCAGAAATG

Sucla2-PR	ACATCCTTTGATCCTGGGCG
Sdha-PF	GCTGTGGCCCTGAGAAAGAT
Sdha-PR	TCCAGGCCTGCAAGATTCTG
Sdhb-PF	CGTTCTCGGCAGAGTCGG
Sdhb-PR	AGACTTTGCTGAGGTCCGTG
Sdhc-PF	AAGATGGCTGCGTTCTTGCT
Sdhc-PR	GGGAGACAGAGGACGGTTTG
Sdhd-PF	GTGACCTTGAGCCCTCGAAA
Sdhd-PR	GCTGGTCCTGGAGAAATGCT
Tnfa-PF	ACCCTCACACTCACAAACCA
Tnfa-PR	ACCCTGAGCCATAATCCCCT
Il1b-PF	TGCCACCTTTTGACAGTGATG
Il1b-PR	TTCTTGTGACCCTGAGCGAC
Arg1-PF	CTCCAAGCCAAAGTCCTTAGAG
Arg1-PR	AGGAGCTGTCATTAGGGACATC
Cd206-PF	GTCAGAACAGACTGCGTGGA
Cd206-PR	AGGGATCGCCTGTTTTCCAG
Tgfb-PF	ACTGGAGTTGTACGGCAGTG
Tgfb-PR	GGGGCTGATCCCGTTGATTT
Nsun2-PF	GCTGCTATCTGCTCGTCCAT
Nsun2-PR	ACGTTTGTTCCACGGCATTG
Eno1-PF	TGCTTTGCAGCGATCCTACT
Eno1-PR	GTGCCGTCCATCTCGATCAT
Isg15-PF	TGGTACAGAACTGCAGCGAG
Isg15-PR	CAGCCAGAACTGGTCTTCGT
Cxcl10-PF	GGTCTGAGTCCTCGCTCAAG
Cxcl10-PR	GTCGCACCTCCACATAGCTT
Bst2-PF	CACAGGCAAACTCCTGCAAC
Bst2-PR	TCCTGGTTCAGCTTCGTGAC

Oas1a-PF	GCTGACCCAACAGGGAATGT
Oas1a-PR	CAGTTCTCTTCTGCTCAAAAGGT
Il6-PF	GCCTTCTTGGGACTGATGCT
Il6-PR	TGTGACTCCAGCTTATCTCTTGG
Actb-PF	TGAGCTGCGTTTTACACCCT
Actb-PR	AAGTCAGTGTACAGGCCAGC
**Primers for RT-qPCR (human genes)**	
CCL2-PF	CAGCCAGATGCAATCAATGCC
CCL2-PR	TGGAATCCTGAACCCACTTCT
CCL5-PF	CCAGCAGTCGTCTTTGTCAC
CCL5-PR	CTCTGGGTTGGCACACACTT
CCL7-PF	CAACTACCTGCTGCTACAGATT
CCL7-PR	CTCCTTGTCCAGTTTGGTCTT
CCL8-PF	TGGAGAGCTACACAAGAATCACC
CCL8-PR	TGGTCCAGATGCTTCATGGAA
CCL20-PF	CCAAGAGTTTGCTCCTGGCT
CCL20-PR	GGATTTGCGCACACAGACAA
CX3CL1-PF	TCCGATATCTCTGTCGTGGC
CX3CL1-PR	TGTCTCGTCTCCAAGCAGC
SUCLG1-PF	AATTCCCCAGCAGGACATGG
SUCLG1-PR	CCAATGCCAACGCACAAAGA
SUCLG2-PF	GAACGTCGCCCACCAAAATC
SUCLG2-PR	TTAGCATGCAGCTGAACCCA
SUCLA2-PF	CAGTGGATGGCTGAAGGTGT
SUCLA2-PR	AGACTGGCTGCGACAAAAGA
SDHA-PF	ACTGTTGCAGCACAGCTAGA
SDHA-PR	GGGGCGTATCGCTCCATAAA
SDHB-PF	GCCCCATGGTATTGGATGCT
SDHB-PR	AGTTGCTCAAATCCTTATTGAGGT

SDHC-PF	GCTGTTGCTGAGACACGTTG
SDHC-PR	CCCTCTAGCTGGGACAGACT
SDHD-PF	AGCTCTGTTGCTTCGAACTC
SDHD-PR	CAACTTGTCCAAGGCCCAATG
ENO1-PF	CACCACCTTGTGGACAGGAA
ENO1-PR	TAAAGCGGGACTGAACACCC
TNFA-PF	GACAAGCCTGTAGCCCATGT
TNFA-PR	GGAGGTTGACCTTGGTCTGG
IL1B-PF	AACCTCTTCGAGGCACAAGG
IL1B-PR	AGATTCGTAGCTGGATGCCG
NOS2-PF	TCCAAGGTATCCTGGAGCGA
NOS2-PR	CAGGGACGGGAACTCCTCTA
ARG1-PF	ACTTAAAGAACAAGAGTGTGATGTG
ARG1-PR	GTCCACGTCTCTCAAGCCAA
CD206-PF	GCCTCGTTGTTTTGCGTCTT
CD206-PR	GAGAACAGCACCCGGAATGA
TGFB1-PF	ACCTGCCACAGATCCCCTAT
TGFB1-PR	CCGGTAGTGAACCCGTTGAT
NSUN2-PF	GCTACCCCGAGATCGTCAAG
NSUN2-PR	TCAGGATACCTTTTGTAACCAGT
ISG15-PF	GTGGACAAATGCGACGAACC
ISG15-PR	ATTTCCGGCCCTTGATCCTG
CXCL10-PF	GCTTCCAAGGATGGACCACA
CXCL10-PR	GCAGGGTCAGAACATCCACT
BST2-PF	GAAGATTCCAGCACCCTCCC
BST2-PR	TTCCATGGGCACTCTGCAAT
IL6-PF	CCACCGGGAACGAAAGAGAA
IL6-PR	GAGAAGGCAACTGGACCGAA
OAS1-PF	GAGCTCCTGACGGTCTATGC

OAS1-PR	CAGTCCTCTTCTGCCTGTGG
ACTB-PF	CATGTACGTTGCTATCCAGGC
ACTB-PR	CTCCTTAATGTCACGCACGAT
**Primers for ChIP qRT-PCR with anti-GATA3 antibody (human genes)**	
ChIP *SUCLG1*-PF	ACATTTCTAGGAAGGAGCCGAG
ChIP *SUCLG1*-PR	GGAATGGCAGGTGAAGACAAA
ChIP *SUCLG2*-PF	TCGGGGTCTGTCTTTGCTCA
ChIP *SUCLG2*-PR	CGGTTCACTTTGAGCGGTCC
ChIP *CCL2*-PF	GGCTATTTAACCCTTCTGTG
ChIP *CCL2*-PR	TCACAGAGTGCTTACTCTGC
ChIP *CCL5*-PF	CGCGCTAGGACAGTGGAATA
ChIP *CCL5*-PR	ACAGCATACTCTATCATTATTGGCT
ChIP *CCL7*-PF	CGAGAGAATGAGTAGCCTGAGC
ChIP *CCL7*-PR	TGCAAGGCTTTGATTCTGGTT
**Primers used in ChIP-qPCR assays with anti-NSUN2 antibody (human genes)**	
ChIP *SUCLG1*-PF-1	GCGACAGCTCAGGAATAATTTTGC
ChIP *SUCLG1*-PR-1	ACAGGTTGGACTTCACTGCA
ChIP *SUCLG1*-PF-2	CCACTCATCCATCCCACAGCT
ChIP *SUCLG1*-PR-2	GTCTCTGCCGAAAATCGTGCT
ChIP *SUCLG1*-PF-3	TGCTCTTCGCACCTCACCTC
ChIP *SUCLG1*-PR-3	CAATGTAGTCTGACGGCCGC
ChIP *SUCLG2*-PF-1	CGCGGTAAAGATGGCGTGAC
ChIP *SUCLG2*-PR-1	CGGACGGGCCACCTTTAAGA
ChIP *SUCLG2*-PF-2	AGGGCTGGCTGGTGTATTTA
ChIP *SUCLG2*-PR-2	AGGAGTTGTGGGTGTTTTGTT
ChIP *SUCLG2*-PF-3	CTGGGGACTGGACAGTAGGC
ChIP *SUCLG2*-PR-3	GGCACAGGCGTTCTTAAGGC
ChIP *FOXA1*-PF-1	CGACGTCCAGGACCGATTAG
ChIP *FOXA1*-PR-1	CTGCACTGCGAAAGAGATGC

ChIP *FOXA1*-PF-2	GCCTGCCCTATCACACAGAC
ChIP *FOXA1*-PR-2	CTCGTACCTGCGGCTAAGAG
ChIP *FOXA1*-PF-3	GCAAACAAAGTGAGGGCCCC
ChIP *FOXA1*-PR-3	GACGCTCGCACCTACAAAGC

Abbreviations: PF, Forward primer; PR, Reverse primer. sgRNA, Single-guide RNA. RT-qPCR, Reverse transcription quantitative real-time polymerase chain reaction. ChIP-qPCR, Chromatin immunoprecipitation coupled with quantitative real-time polymerase chain reaction; Actb, Actin beta; Arg1, Arginase 1; Bst2, Bone marrow stromal cell antigen 2; Ccl2, C-C motif chemokine ligand 2; Ccl5, C-C motif chemokine ligand 5; Ccl7, C-C motif chemokine ligand 7; Ccl8, C-C motif chemokine ligand 8; Ccl20, C-C motif chemokine ligand 20; Cx3cl1, C-X3-C motif chemokine ligand 1; Cxcl10, C-X-C motif chemokine ligand 10; Eno1, Enolase 1; FOXA1, Forkhead box A1; Gata3, GATA binding protein 3; Il1b, Interleukin 1 beta; Il6, Interleukin 6; Isg15, Interferon-stimulated gene 15; NOS2, Nitric oxide synthase 2; Nsun2, NOP2/Sun RNA methyltransferase 2; Oas1a, 2'-5'-oligoadenylate synthetase 1A; Sdha, Succinate dehydrogenase complex flavoprotein subunit A; Sdhb, Succinate dehydrogenase complex iron sulfur subunit B; Sdhc, Succinate dehydrogenase complex subunit C; Sdhd, Succinate dehydrogenase complex subunit D; Sucla2, Succinyl-CoA ligase alpha subunit 2; Suclg1, Succinyl-CoA ligase gamma subunit 1; Suclg2, Succinyl-CoA ligase gamma subunit 2; Tgfb/TGFB1, Transforming growth factor beta 1; Tnfa/TNFA, Tumor necrosis factor alpha.

Supplementary Table S2. Antibodies used in this study.

Assays	Antibodies	Vendors	Catalog No.	Working dilution
WB	anti-phospho-STING (Ser365/366)	Cell signaling technology	50907T	1:1000
	anti-STING	ABclonal	A21051	1:2000

	anti-phospho-TBK1 (Ser172)	ABclonal	AP1026	1:1000
	anti-TBK1	ABclonal	A3458	1:2000
	anti-phospho-IRF3 (Ser396)	Cell signaling technology	4947S	1:1000
	anti-IRF3	ABclonal	A19717	1:2000
	anti-Flag	ABclonal	AE005	1:3000
	anti-HA	Proteintech	51064-2-AP	1:3000
	anti-SUCLG1	Proteintech	14923-1-AP	1:3000
	anti-SUCLG2	Proteintech	14240-1-AP	1:3000
	anti-NSUN2	Proteintech	20854-1-AP	1:3000
	anti-NSUN5	Proteintech	15449-1-AP	1:3000
	anti-NSUN6	Proteintech	15449-1-AP	1:3000
	anti-ENO1	Proteintech	11204-1-AP	1:4000
	anti-GATA3	Proteintech	66400-1-Ig	1:1500
	anti-p65	Proteintech	80979-1-RR	1:2000
	anti-p50	Proteintech	14220-1-AP	1:2000
	anti-Relb	Proteintech	83305-6-RR	1:2000
	anti-H3	Proteintech	17168-1-AP	1:5000
	anti-GAPDH	Proteintech	60004-1-Ig	1:5000
Immunoprecipitation	anti-Flag	ABclonal	AE005	3 μL/test
	anti-HA	Proteintech	51064-2-AP	3 μL/test
	anti-NSUN2	Proteintech	20854-1-AP	3 μL/test
	anti-NSUN5	Proteintech	15449-1-AP	3 μL/test
	anti-NSUN6	Proteintech	15449-1-AP	3 μL/test
	anti-GATA3	Proteintech	66400-1-Ig	5 μL/test
Immunofluorescence	anti-NSUN2	Proteintech	20854-1-AP	1:200
	anti-GATA3	Proteintech	66400-1-Ig	1:100

Flow cytometry	anti-CD45	BioLegend	103105	3 μL/test
	anti-CD45	BD Pharmingen	560510	3 μL/test
	anti-CD3	BioLegend	100236	3 μL/test
	anti-CD4	BioLegend	100412	3 μL/test
	anti-CD8	BioLegend	100705	3 μL/test
	anti-CD8	abcam	ab217344	3 μL/test
	anti-CD11b	BioLegend	101205	3 μL/test
	anti-CD11b	BioLegend	101215	3 μL/test
	anti- F4/80	BioLegend	123109	3 μL/test
	anti-CD206	BioLegend	141709	3 μL/test
	anti-ARG1	eBioscience	48-3697-82	3 μL/test
	anti-CD86	BioLegend	10511	3 μL/test
	anti-Nos2 (iNOS)	eBioscience	61-5920-82	3 μL/test
	anti-IFN-γ	BioLegend	505808	3 μL/test
	anti-GzmB	BioLegend	372209	3 μL/test

Abbreviations: WB, Western blot; IP, Immunoprecipitation; IF, Immunofluorescence; FCM, Flow cytometry; Phospho-, phosphorylated protein; STING, Stimulator of interferon genes; TBK1, TANK-binding kinase 1; IRF3, Interferon regulatory factor 3; SUCLG1, Succinyl-CoA ligase gamma subunit 1; SUCLG2, Succinyl-CoA ligase beta subunit 2; NSUN2, NOP2/Sun RNA methyltransferase 2; NSUN5, NOP2/Sun RNA methyltransferase 5; NSUN6, NOP2/Sun RNA methyltransferase 6; ENO1, Enolase 1; GATA3, GATA binding protein 3; p65, NF-κB p65 subunit; p50, NF-κB p50 subunit; RelB, RELB proto-oncogene; H3, Histone H3; GAPDH, Glyceraldehyde-3-phosphate dehydrogenase; F4/80, EGF-like module-containing mucin-like hormone receptor-like 1; ARG1, Arginase 1; NOS2 (iNOS), Inducible nitric oxide synthase 2; IFN-γ, Interferon gamma; GzmB, Granzyme B.
